# Single-Arm, Multicenter Phase I/II Clinical Trial for the Treatment of Envenomings by Massive Africanized Honey Bee Stings Using the Unique Apilic Antivenom

**DOI:** 10.3389/fimmu.2021.653151

**Published:** 2021-03-23

**Authors:** Alexandre Naime Barbosa, Rui Seabra Ferreira, Francilene Capel Tavares de Carvalho, Fabiana Schuelter-Trevisol, Mônica Bannwart Mendes, Bruna Cavecci Mendonça, José Nixon Batista, Daisson José Trevisol, Leslie Boyer, Jean-Philippe Chippaux, Natália Bronzatto Medolago, Claudia Vilalva Cassaro, Márcia Tonin Rigotto Carneiro, Ana Paola Piloto de Oliveira, Daniel Carvalho Pimenta, Luís Eduardo Ribeiro da Cunha, Lucilene Delazari dos Santos, Benedito Barraviera

**Affiliations:** ^1^ Department of Infectology, Dermatology, Imaging Diagnosis and Radiotherapy, Botucatu Medical School (FMB), São Paulo State University (UNESP – Univ Estadual Paulista), Botucatu, Brazil; ^2^ Graduate Program in Tropical Diseases, Botucatu Medical School (FMB), São Paulo State University (UNESP—Univ Estadual Paulista), Botucatu, Brazil; ^3^ Center for the Study of Venoms and Venomous Animals (CEVAP), São Paulo State University (UNESP—Univ Estadual Paulista), Botucatu, Brazil; ^4^ Graduate Program in Clinical Research, Center for the Study of Venoms and Venomous Animals (CEVAP) and Botucatu Medical School (FMB), São Paulo State University (UNESP—Univ Estadual Paulista), Botucatu, Brazil; ^5^ Clinical Research Center, Nossa Senhora da Conceição Hospital, Tubarão, Brazil; ^6^ Graduate Program in Health Sciences, University of Southern Santa Catarina at Tubarão, Tubarão, Brazil; ^7^ VIPER Institute, University of Arizona College of Medicine, Tucson, AZ, United States; ^8^ MERIT, IRD, Université Paris 5, Sorbonne Paris Cité, Paris, France; ^9^ CRT, Institut Pasteur, Paris, France; ^10^ Clinical Research Unit (UPECLIN), Botucatu Medical School, São Paulo State University (UNESP—Univ Estadual Paulista), Botucatu, Brazil; ^11^ Biochemistry and Biophysics Laboratory, Butantan Institute, São Paulo, Brazil; ^12^ Antivenom Production Laboratory, Vital Brazil Institute, Rio de Janeiro, Brazil

**Keywords:** antivenom, *Apis mellifera* (Africanized), clinical trial, safety assessment, enzyme-linked immunosorbent assay (ELISA)

## Abstract

We evaluated the safety, optimal dose, and preliminary effectiveness of a new-approach Africanized honeybee (*Apis mellifera*) Antivenom (AAV) in a phase I/II, multicenter, non-randomized, single-arm clinical trial involving 20 participants with multiple stings. Participants received 2 to 10 vials of AAV depending on the number of stings they suffered, or a predefined adjuvant, symptomatic, and complementary treatment. The primary safety endpoint was the occurrence of early adverse reactions within the first 24 h of treatment. Preliminary efficacy based on clinical evolution, including laboratory findings, was assessed at baseline and at various time points over the four following weeks. ELISA assays and mass spectrometry were used to estimate venom pharmacokinetics before, during, and after treatment. Twenty adult participants, i.e., 13 (65%) men and 7 (35%) women, with a median age of 44 years and a mean body surface area of 1.92 m^2^ (median = 1.93 m^2^) were recruited. The number of stings ranged from 7 to > 2,000, with a median of 52.5. Symptoms of envenoming were classified as mild, moderate, or severe in 80% (16), 15% (3), and 5% (1) of patients, respectively; patients with mild, moderate, or severe envenoming received 2, 6, and 10 vials of AAV as per the protocol. None of the patients had late reactions (serum sickness) within 30 d of treatment. There was no discontinuation of the protocol due to adverse events, and there were no serious adverse events. One patient had a moderate adverse event, transient itchy skin, and erythroderma. All participants completed the intravenous antivenom infusion within 2 h, and there was no loss to follow-up after discharge. ELISA assays showed venom (melittin and PLA_2_) concentrations varying between 0.25 and 1.479 ng/mL prior to treatment. Venom levels decreased in all patients during the hospitalization period. Surprisingly, in nine cases (45%), despite clinical recovery and the absence of symptoms, venom levels increased again during outpatient care 10 d after discharge. Mass spectrometry showed melittin in eight participants, 30 d after treatment. Considering the promising safety results for this investigational product in the treatment of massive Africanized honeybee attack, and its efficacy, reflected in the clinical improvements and corresponding immediate decrease in blood venom levels, the AAV has shown to be safe for human use. **Clinical Trial Registration:** UTN: U1111-1160-7011, identifier [RBR-3fthf8].

## Introduction

In 1956, African honeybees of the subspecies, *Apis mellifera scutellata*, were introduced from Tanganyika and South Africa to Brazil because they were more productive and resistant to pests (1). They incidentally escaped and crossed with the existing European bees of the subspecies, *Apis mellifera mellifera*, resulting in an Africanized hybrid. These presented marked defensive and swarming capacities, and easily adapted to different climates and environments. In addition, these capacities made it possible for them to expand throughout Brazil and several other countries in the Western Hemisphere, including the United States (2–4). When threatened, these honeybees massively attack the target; consequently, the number of accidents involving humans and animals has increased (3, 5, 6).

Bee venom is composed of a complex mixture of biogenic amines, proteins, enzymes, and peptides. Among these are proteins of low allergenic importance and intense pharmacological action such as melittin, phospholipase A_2_, apamine, hyaluronidase, and several low molecular weight peptides, which constitute approximately 50–60%, 11–12%, 3%, 1–2%, and 1% of the gross weight, respectively, and water, and mineral salts.

Melittin and phospholipase A_2_ are the two most toxic components of the bee venom and may act synergistically to induce a variety of pathophysiological effects (7–10).

**Figure 1:**
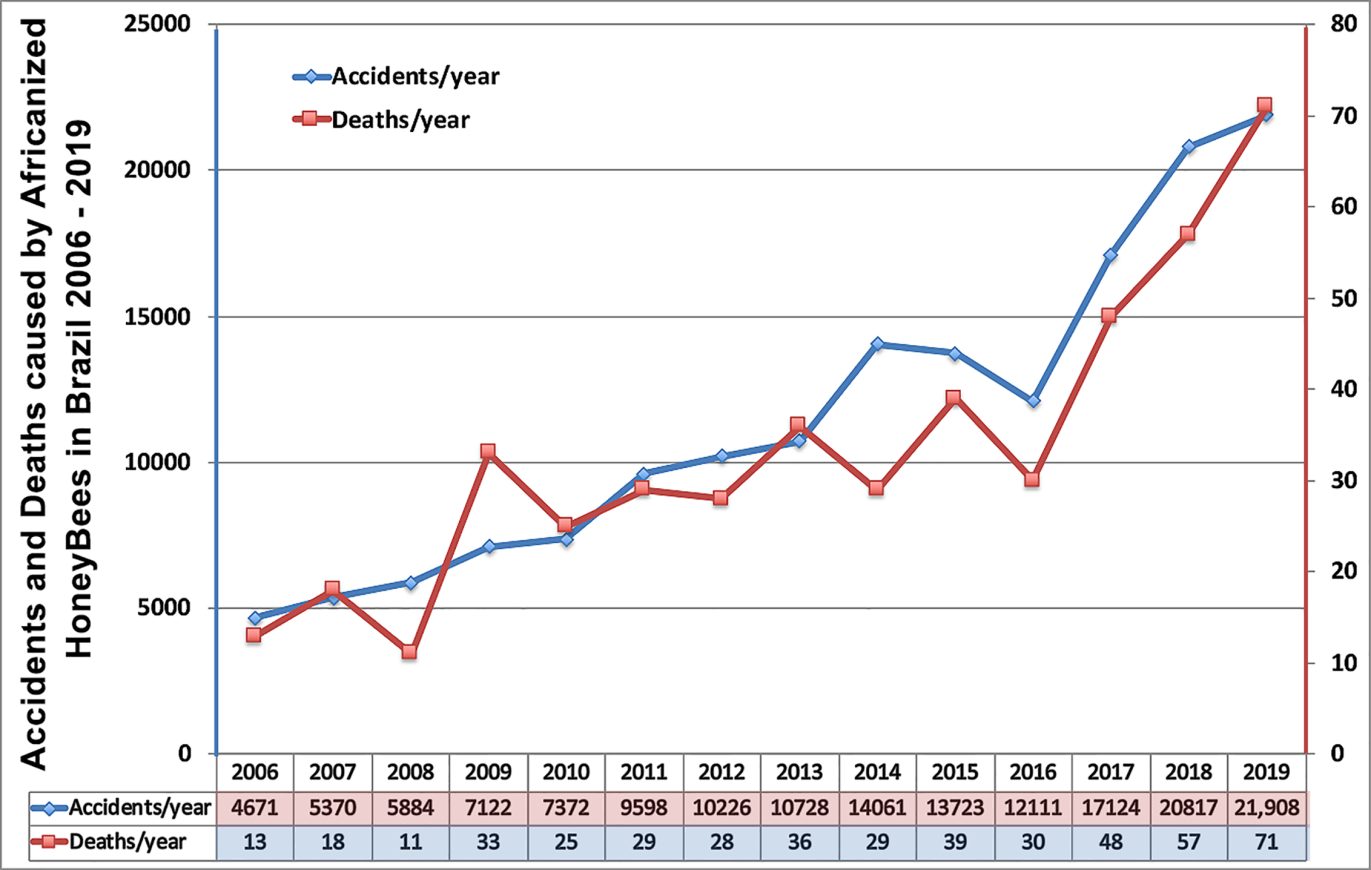
Annual distribution of the number of accidents and deaths caused by AHB in Brazil between 2006 and 2019 (14).

Envenoming by venomous snakes is a serious public health problem in tropical countries and is one of the most neglected health problems according to the World Health Organization’s (WHO) classification (11, 12). Health effects become more serious when the envenoming is caused by the injection of fairly large amounts of venom, such as that caused by the bites of adult specimens of *Bothrops jararaca*. Similarly, *A. mellifera* attacks massively, and thousands of bees can inject up to half a gram of venom. The expansion of Africanized honeybees (AHB) throughout the Americas (3, 4, 13), and consequently, the severity of the accidents they provoke, led health authorities to classify such accidents as objects of health surveillance in Brazil (**Figure 1**) (14).

These accidents show different magnitudes of clinical manifestation depending mainly on the number of stings and the individual’s sensitivity. The most frequent accident is that involving individuals not sensitized to the venom, affected by a few stings. In these cases, the clinical presentation is limited to a local inflammatory reaction, which manifests as erythematous papules, pain, and heat. In most cases, this situation is resolved without medical intervention. Another clinical form is that involving individuals sensitized to one or more components of the venom; they exhibit an immediate type I hypersensitivity reaction, as defined by Coombs and Gell (15). This serious event can be triggered by a single sting, and requires immediate medical intervention. Generally, the clinical manifestations include glottal edema, angioedema, and bronchospasm associated with anaphylactic shock (3, 5, 6).

The third presentation is that caused by multiple stings occurring when an individual is attacked by a swarm of honeybees. Here, a large amount of venom is inoculated, usually caused by hundreds or thousands of honeybees (3, 5, 6). These patients present with generalized pain, intense itching, and agitation, which may progress to numbness, associated with severe acute respiratory and kidney failure. Patients who die present on pathological examination acute tubular necrosis, with the presence of heme and/or myoglobin casts inside the renal tubules or glomeruli. There is usually intense proteolysis in skeletal muscles, with the release of myoglobin and creatine kinase (CK) into the blood. The heart may be affected, in which case a sub-endocardial lesion with infarction is observed. The liver may show signs of hydropic degeneration in cases of severe envenoming (16–18).

Laboratory findings change rapidly and the blood cell count may show leukocytosis with neutrophilia and a staggered leftward shift, and type I urinalysis usually reveals proteinuria, glycosuria, and the presence of heme pigments. Serum urea and creatinine levels may increase due to kidney damage. Levels of CK and aspartate aminotransferase (AST) are usually elevated due to severe rhabdomyolysis. Alanine aminotransferase (ALT) levels may increase over time, indicating liver failure. Finally, levels of acute-phase proteins, including the C-reactive protein (CRP) and fibrinogen, are usually altered, indicating a severe systemic inflammatory response syndrome (3, 5, 6, 18).

Until recently, the treatment of patients who suffered from both smaller and larger numbers of stings was symptomatic and relied on antihistamines, corticosteroids, and even epinephrine in case of anaphylactic shock. The search for a specific treatment based on heterologous antivenoms has been a challenge for many researchers (19, 20). In 2000, Ferreira Jr. et al. (3) initiated the development of a new antivenom constituted only of antibodies against the two main toxins, melittin and phospholipase A_2_ (8, 9, 21). In 2017, Barbosa et al. (22) published a clinical protocol for the treatment of patients with multiple stings, which was applied in the present clinical study. Recently, Teixeira-Cruz et al. (23) published preclinical results, focusing mainly on the neutralization of biochemical and pharmacological activities of bee venom by apilic antivenom and they have concluded that this specific antivenom emerges as a new promising immunobiological product for the treatment of massive AHB attacks.

This clinical study was performed to assess the safety, establish the optimal minimum dose, and evaluate the preliminary effectiveness of the novel Apilic Antivenom (AAV).

## Patients and Methods

### Ethical Approval and Consent to Participate

The clinical protocol was approved by the Brazilian National Commission on Ethics in Research (CONEP, Certificate of Presentation of Ethical Appreciation No. 19006813.4.1001.5411, v7, approved on 06/07/2016), and the Brazilian National Health Surveillance Agency (ANVISA), which approved the Apis Study on 02/05/2016; No. 0907532142; Proc. No. 25361611582201493. This trial RBR-3FTHF8 was registered in 2015 in the Brazilian Clinical Trials Registry (ReBEC) (24). The first participant was recruited on 08/22/2016; Universal Trial Number (UTN): U1111-1160-7011; Register Number: RBR-3fthf8; Public access URL: http://www.ensaiosclinicos.gov.br/rg/RBR-3fthf8/. The clinical trial protocol was published by Barbosa et al. (22).

### Study Design

**Figure 2:**
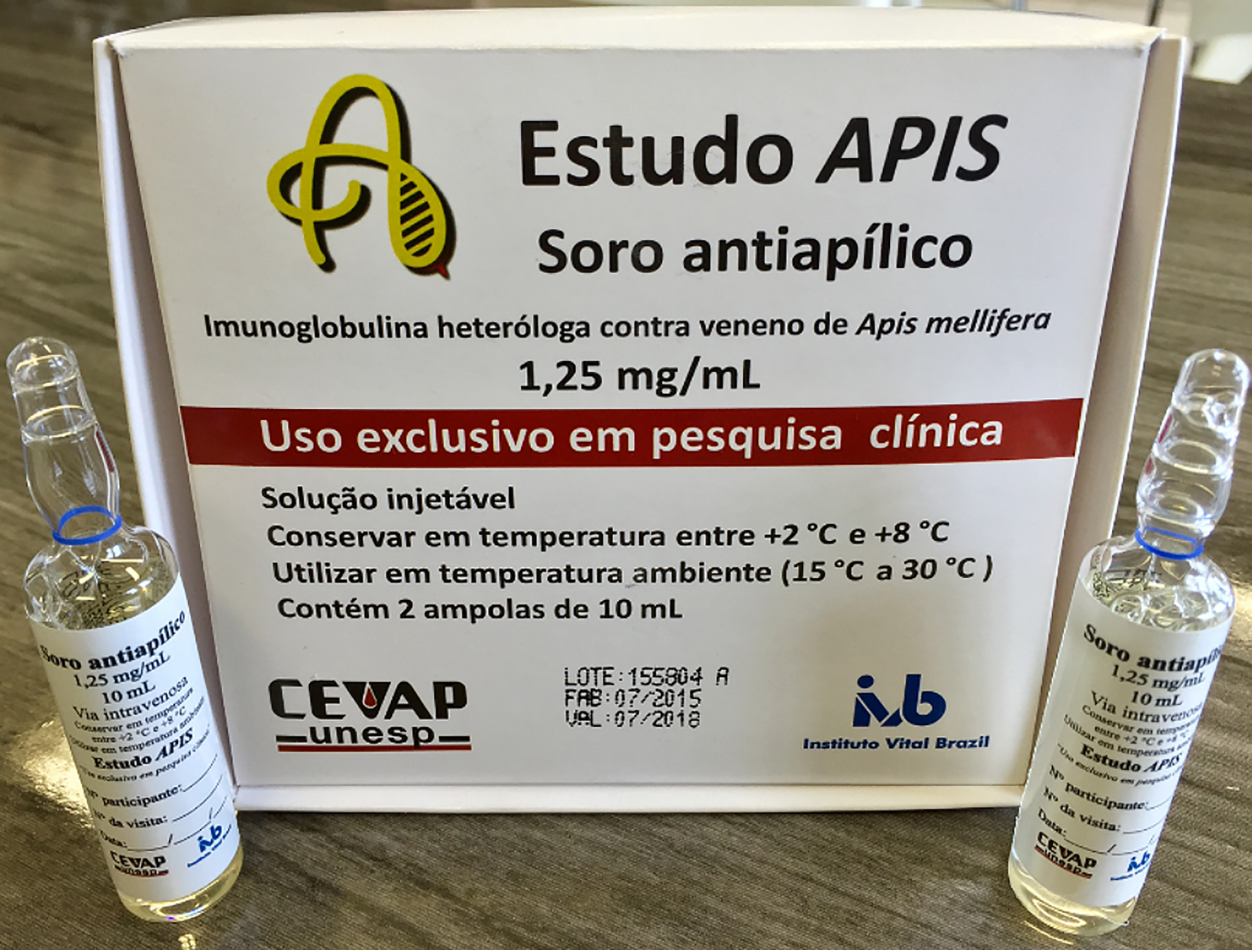
New Apilic Antivenom.

This was a phase I/II, multicenter, non-randomized, single-arm clinical trial study, involving 20 AHB multiple-sting participants treated with the new Apilic Antivenom “*batch 155804 R*” (**Figure 2**), performed from 08/22/2016 to 07/27/2018, in which 1 mL of AAV neutralized 1.25 mg of whole honeybee venom (23). Two clinical research units of the Brazilian National Clinical Research Network (RNPC) were used for this study; these were the Botucatu Medical School, São Paulo State University, São Paulo, Brazil, and the Clinical Research Center of the Nossa Senhora da Conceição Hospital (HNSC), Tubarão, Santa Catarina, Brazil. All participants provided informed consent by signing the free and informed consent form (FICF).

### Outcomes

The primary endpoints were to assess the safety of AAV, based on the occurrence of adverse events, in participants exposed to multiple Africanized honeybees’ stings, and to estimate the proportion of participants showing improvements with respect to their initial clinical state, by monitoring symptoms and laboratory test results.

The secondary endpoint was to determine the correlation between the number of stings and the severity of the initial clinical presentation using the AHB score, adapted for this envenoming, based on APACHE II (25).

In addition, an exploratory endpoint was included in the study to estimate the pharmacokinetic profile of the AHB venom using ELISA tests carried out on blood samples collected at different time points (before AVV administration and 2, 6, 12, and 24 h after AAV administration). Furthermore, we aimed to assess the acute-phase reaction profile of AAV, by monitoring variations in C-reactive protein (CRP) and fibrinogen levels.

### Inclusion, Exclusion, and Discontinuation Criteria

All participants who presenting with AHB stings were screened for eligibility by the clinical staff (**Table 1**). People aged over 18 years were eligible to receive AAV with respect to the number of stings, as described below. Each participant or his/her relative signed the FICF. **Supplementary Video 1** - Stringer Video shows the procedure for removing the sting of Africanized bees in one of the patients.

**Table 1:** Inclusion, exclusion, and discontinuation criteria.

Inclusion	Exclusion	Discontinuation
Participants over 18 years of age of both sexes	Participants who had a previous adverse reaction to heterologous serum produced in horses.	Developing anaphylactic shock resistant to the management protocol for reactions of acute hypersensitivities.
Participants admitted to the hospital after an accident with AHB	Participants who are pregnant or nursing	Withdrawing from the terms of free and informed consent form (FICF)
Participants or a responsible relative who signs informed consent (FICF) to receive the antivenom.	–	–

### Antivenom Doses and Adjuvant Treatments

Before treatment, height in centimeters (cm), body weight in kilograms (kg), body mass index in Kg/m^2^; and body surface area in square meters (m^2^) were measured, as described by Madden and Smith (26). Then, all participants received a single intravenous administration of the AAV, diluted in a solution of 250 mL of 0.9% sodium chloride intravenously over two h, according to the protocol, and based on the number of stings as described below:

- Up to 5 stings: Specific treatment with AAV was not indicated. Here, only adjuvant, symptomatic, and complementary treatments were administered.
- Between 5 and 200 stings: Two vials (20 mL) of AAV;
- Between 201 and 600 stings: Six vials (60 mL) of AAV;
- Above 600 stings: Ten vials (100 mL) of AAV.

The adjuvant, symptomatic, and complementary treatments were described and published in detail by Barbosa et al. (22) at http://www.doi:10.1186/s40409-017-0106-y.

### Criteria for Measuring the Severity and Clinical Outcomes in the Participants

An AHB score was assigned based on APACHE II, which is a validated system for classifying disease severity (25). The proposed AHB score varied between 1 and 15 according to the severity criteria, which included eight clinical and seven strategic laboratory alterations, based on medical literature, assessed at the time of the first examination (4, 7, 16–18). These criteria, except for those that did not change throughout the treatment period (i.e., age > 60 years, body mass index over 30 kg/m^2^, time elapsed between the accident and medical care, and number of stings) were evaluated and monitored throughout the course of the treatment to verify clinical outcomes, and the normalization of laboratory parameters upon discharge from the hospital and during follow-up visits, 10, 20, and 30 d later was also monitored.

### AHB Score Proposed for Clinical and Laboratory Assessments

-Age > 60 years: Score 1

-Body mass index > 30 kg/m^2^: Score 1

-Time elapsed between the accident and medical care (over 24 hours): Score 1

-Number of stings

- 5–200 stings: Score 1
- 201–600 stings: Score 2
- More than 600 stings: Score 3

-Hemodynamic disorders (tachycardia, arterial hypotension, shock): Score 1

-Respiratory disorders (bradypnea, bronchospasm, wheezing, and/or dyspnea): Score 1

-Neurological disorders (mental confusion and/or intense headache): Score 1

-Acute kidney injury (anuria and/or oliguria): Score 1

-Increased CK levels: Score 1

-Increased ALT levels: Score 1

-Increased creatinine levels: Score 1

-Increased CRP levels: Score 1

-Increased fibrinogen levels: Score 1

-Increased leukocyte levels: Score 1

-Decreased platelet count: Score 1

### Subsidiary and Strategic Laboratory Tests

Laboratory tests (CK, ALT, creatinine, CRP, fibrinogen, leukocytes, and platelets) were used to assess safety parameters before treatment (hospital admission), on the day of discharge, and at follow-up (10, 20, and 30 d after discharge from the hospital). All procedures and protocols for laboratory tests are presented in **Data Sheet 1-Tables-1-2** (27, 28).

### Classification of Early and Late Adverse Reactions to Antivenom

Adverse reactions to AAV were defined as mild, moderate, or severe based on the international classification of anaphylactic reactions described in **Table 2** (29, 30). During the outpatient evaluations (10, 20, and 30 d), a clinical history focused on serum sickness (a type III hypersensitivity reaction) was taken. Thus, the patient was asked if in the 10 d prior to the consultation he had had any type of cutaneous rash, fever, lymphadenopathy, facial and periorbital edema, changes in urine color, and/or arthralgia.

**Table 2:** Classification of acute adverse reactions to antivenoms (29, 30).

Mild	Moderate	Severe
Facial edema	Abdominal pain	Drowsiness or altered consciousness
Pruritus	Nausea	Systolic BP < 80 mm Hg
Urticaria	Vomiting	Cyanosis
Fever	Bronchospasm	Confusion
Rigor	Stridor	Shock

### ELISA Assays for the Estimation of Venom Pharmacokinetics

F(ab’)_2_-type immunoglobulin fractions prepared from the blood of hyperimmunized horses against melittin and PLA_2_, found in the venom of *A. mellifera* [Anti-melittin F(ab’)_2_, and Anti-PLA_2_ F(ab’)_2_], were obtained from the AAV using a modified version of the single affinity chromatographic step described by Chávez-Olórtegui et al. (31).

Crude venom (32 mg) from *A. mellifera* was immobilized with 3 g of CNBr-Sepharose resin and prepared according to the manufacturer’s instructions (Cytiva GE Healthcare Life Sciences - USA) for the aforementioned affinity chromatography assay. Anti-melittin F(ab’)_2_ and Anti-PLA_2_ F(ab’)_2_ were conjugated to peroxidase (HRP - Sigma) according to the method described by Nakane and Kawoi (32). The conjugate was titrated as described by Chávez-Olórtegui et al. (31), modified with the conjugate diluted at ratios of 1:20, 1:2,000, 1:5,000, and 1:10,000, and its viability was evaluated for 30 min.

Then, an ELISA assay was performed to quantify the melittin and PLA_2_ fractions in the blood of participants by the method described by Bucaretchi et al. (33). For this, 96-well plates were sensitized with 100 μL of an anti-melittin F(ab’)_2_ and anti- PLA_2_ F(ab’)_2_ mixture at a concentration of 20 μg/mL. The conjugate was used at a dilution of 1:2,000; both in the calibration curve of the *A. mellifera* crude venom and that of the blood samples of the participants. Calculations were performed using the Microsoft Excel software v.16, using linear regression analysis and dose-response curves. The results were expressed in ng/mL.

### Mass Spectrometry Analyses

The participants’ sera (50 µL) were added to 5% DMSO, 0.1% acetic acid (50 µL), and vortexed for 30 min. Then, the solution was centrifuged for 3 min at 3000 *× g*. The supernatant was collected for further processing. Purified melittin from crude *A. mellifera* venom (8) was used as a standard for method development.

Samples were analyzed by liquid chromatography-tandem mass spectrometry in an ESI-IT-TOF instrument coupled to a UFLC 20A Prominence (Shimadzu, Kyoto, Japan). Samples (15 μL) were injected into a C18 column (Kinetex C18 2.6 µm 100 Å, 100 × 4.6 mm), and analyzed using a binary gradient, employing the following solvents: (A) water:DMSO:acetic acid (949:50:1) and (B) ACN:DMSO:water:acetic acid (850:50:99:1). Optimal detection conditions for melittin were achieved at an elution gradient of 25–50% B for 20 min at a constant flow of 0.7 mL.min^-1^, after initial isocratic elution for 5 min. Eluates were monitored using a Shimadzu SPD-M20A PDA detector (Shimadzu, Kyoto, Japan) before being injected into the mass spectrometer.

**Figure 3:**
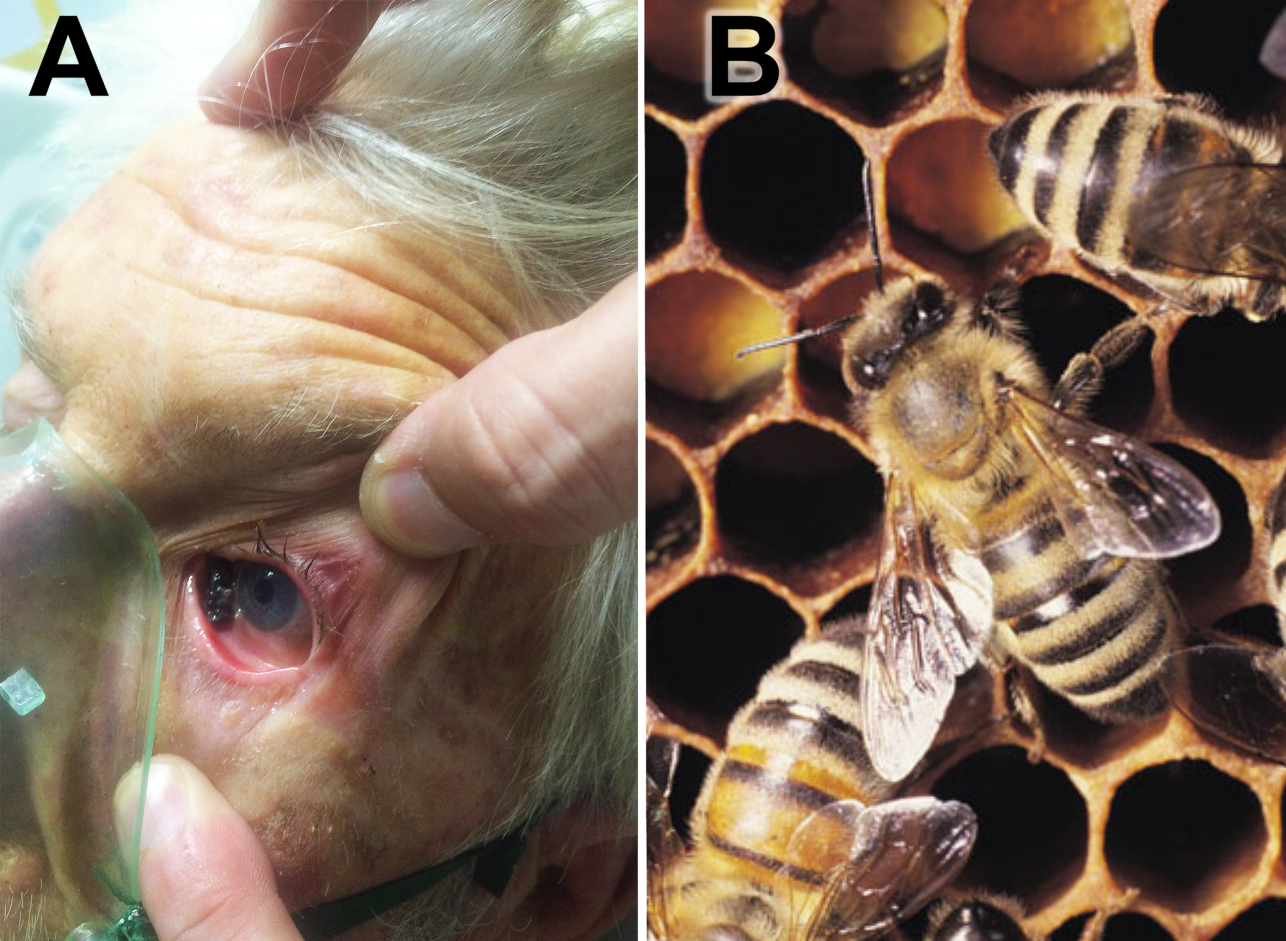
**(A)** Participant 302 with *A*. *mellifera* Africanized honeybee inside the ocular conjunctiva. **(B)** *Apis mellifera* Africanized honeybee.

**Figure 4:**
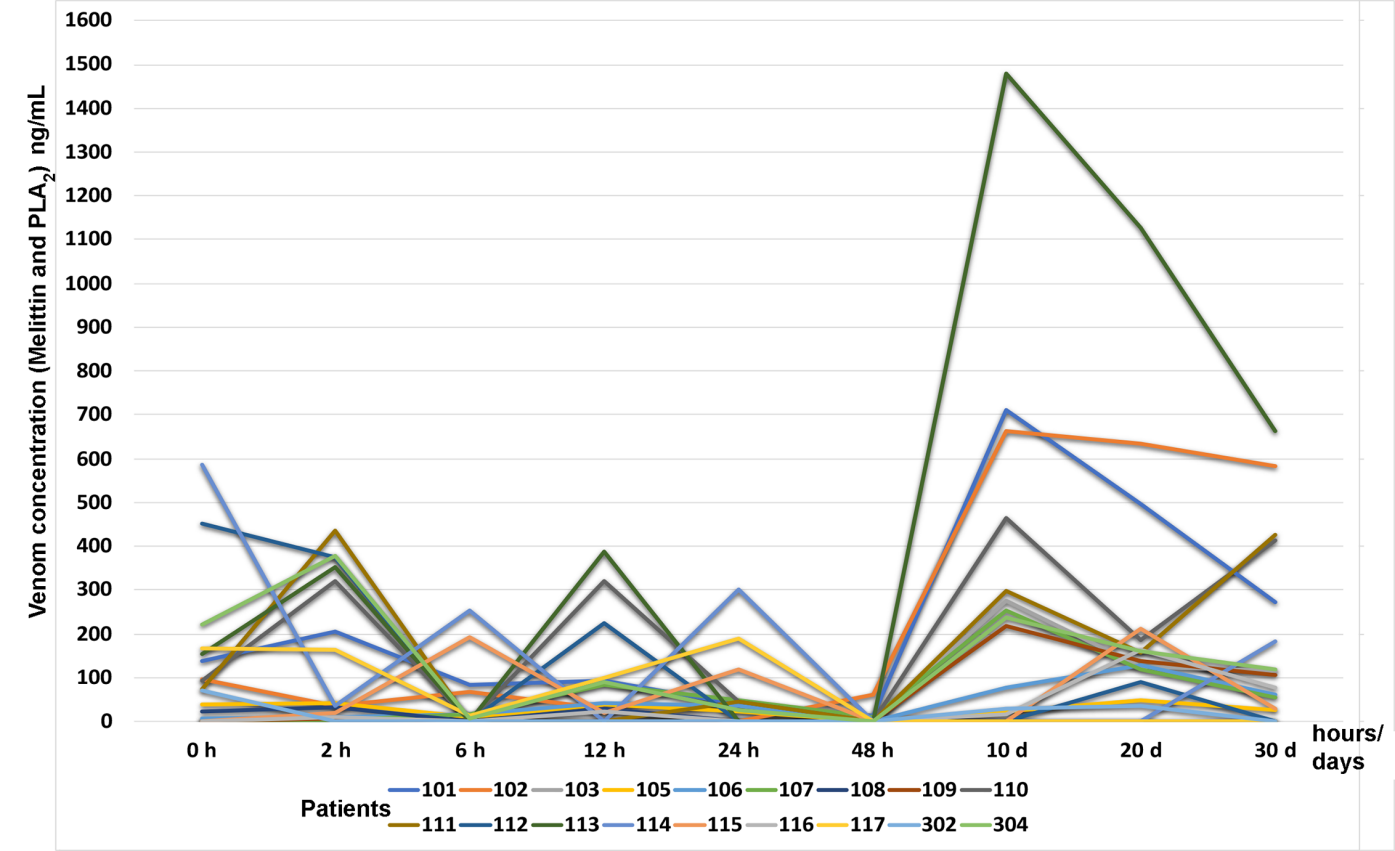
Time-course of melittin + phospholipase A_2_ levels (ng/mL) by ELISA test in eighteen participants at admission, 2, 6, 12, 24, and 48 h after admission and AAV administration, and at 10, 20, and 30 d during the outpatient follow-up period.

**Figure 5:**
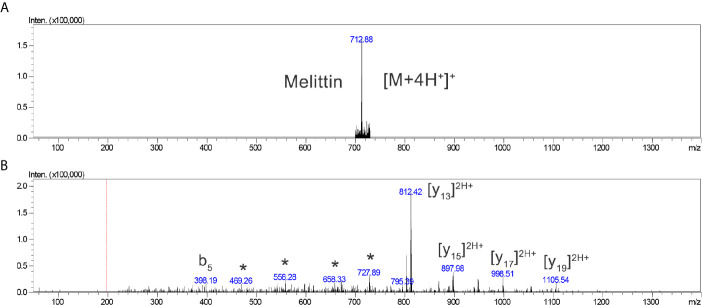
Representative mass spectrometry profile for the qualitative assessment of the presence of melittin in the serum of participant 115, considered positive (+++): **(A)** Melittin [M+4H]4+ MS profile and **(B)** MS^2^ interpreted profile, annotated for the larger b and y ions, as well as internal fragments (*).

The interface was maintained at 4.5 kV and 275°C. The detection voltage was 1.95 kV, and fragmentation was induced using argon collision, with 55 ‘energy’ parameters. MS spectra were acquired in positive mode in the 700–730 m/z range, and MS/MS spectra were obtained in the 50–1400 m/z range, according to the previous optimization with purified melittin. The m/z ion, 712.15 (M+4H^+^), was selected for fragmentation, and the [y_13_]^2+^ ion (811.95, the tallest peak) was monitored at MS^2^ (**Data Sheet 2 - Figures 1-5**).

### Statistical Analysis

Statistical analyses, and the choice comparison tests used among the research participants, were carried out with respect to presuppositions determined by the results, characteristics, and course of the variables in the study. Binomial variables were compared using the chi-squared and Fisher’s exact tests. Numerical values were compared using the Student’s *t*-test or the Mann–Whitney U test. Statistical analyses of the pharmacokinetic assays were performed using the GraphPad Prism software version 8.3.0, with differences considered to be statistically significant when p < 0.05. The results obtained were compared using the ANOVA test for repeated measures, followed by Tukey’s test. The data are represented as the mean ± standard error of the mean (34, 35).

## Results

### Description of Participants

Twenty participants were included, i.e., 13 males (65%) and 7 females (35%). Participants’ ages varied between 22 and 77 years, with a median age of 44 years. Nineteen patients were white-skinned, and one was brown-skinned. Sixteen of them were from the Botucatu region (SP) and four from Tubarão (SC). The number of stings varied from 7 to more than 2,000. The number of vials of AAV administered, based on the clinical protocol, was as follows: mild cases (two vials), 16 participants; moderate cases (six vials), 3 participants; and severe cases (10 vials), 1 participant. The time elapsed between the accident and the clinical care varied as follows: less than 24 h, 5 cases; 1 d, 5 cases; and between 2 and 6 d, 8 cases. One participant was attended 10 and another 19 days after the accident (**Table 3**).

**Table 3:** Description of participants; study protocol number, age, sex, clinical care location, occupation, the estimated number of stings, number of AVV vials administered, and the time elapsed between the accident and medical care in days.

Protocol number	Age	Sex	Clinical care location	Occupation	Estimated number of stings	Number of antivenom vials	Time between accident and clinical care
00101	32	F	Botucatu	B	400	6	3
00102	32	M	Botucatu	B	40	2	10
00103	38	M	Botucatu	A	10	2	0
00105	23	M	Botucatu	A	16	2	0
00106	30	F	Botucatu	A	10	2	2
00107	54	F	Botucatu	A	150	2	1
00108	52	M	Botucatu	A	500	6	19
00109	49	M	Botucatu	A	55	2	2
00110	46	M	Botucatu	A	165	2	2
00111	30	F	Botucatu	A	10	2	4
00112	50	F	Botucatu	B	30	2	4
00113	42	F	Botucatu	B	50	2	4
00114	36	M	Botucatu	A	500	6	1
00115	61	M	Botucatu	B	100	2	1
00116	34	M	Botucatu	A	180	2	1
00117	46	M	Botucatu	A	2000	10	6
00301	61	M	Tubarão	A	20	2	0
00302	77	M	Tubarão	A	150	2	0
00303	22	M	Tubarão	A	7	2	1
00304	66	F	Tubarão	A	50	2	0

M, male; F, female; HCFMB, Hospital of Clinics at Botucatu Medical School (UNESP). HNSC, Nossa Senhora da Conceição Hospital at Southern Santa Catarina University (UNISUL), Tubarão (SC); Occupation: A – occupation not related to agricultural or wilderness activities; B – occupation related to agricultural or wilderness activities; Colors: white – mild cases; yellow – moderate cases; orange – severe case.

As shown in **Table 4**, height varied between 151 and 190 cm, body mass index (BMI) ranged from 19.3 to 32.8 kg/m^2^ (mean: 26.4; median: 26.1), and body surface area (BSA) varied from 1.54 to 2.30 m^2^ (mean: 1.92 m^2^; median: 1.93 m^2^).

**Table 4:** Description of participants; study protocol number, height in centimeters (cm), weight in kilograms (Kg), body mass index in Kg/m^2^, body surface area (m^2^), and estimated number of stings.

Protocolnumber	Height in centimeters(cm)	Weight in kilograms(Kg)	Body mass index	Body surface area	Estimated number of stings
00101	176	65	21.0	1.78	400
00102	175	80	26.1	1.97	40
00103	178	94	29.7	2.16	10
00105	190	100	27.7	2.30	16
00106	157	62,8	25.5	1.66	10
00107	165	81	29.8	1.93	150
00108	175	77	25.1	1.93	500
00109	179	77	24.0	1.96	55
00110	165	52,5	19.3	1.54	165
00111	168	70	24.8	1.81	10
00112	161	67	25.8	1.73	30
00113	175	88	28.7	2.07	50
00114	188	98,1	27.8	2.26	500
00115	167	74	26.5	1.85	100
00116	182	78	23.5	1.99	180
00117	173	89	29.7	2.07	2000
00301	198	–	–	–	20
00302	168	70	24.8	1.81	150
00303	167	91,5	32.8	2.05	7
00304	151	68	29.8	1.69	50

1-Body mass index in Kg/m^2^ (BMI), estimated body surface area (BSA) in m^2^. Colors: White – mild cases; Yellow – moderate cases; orange – severe cases.

Participant 302, aged 77, received 150 stings and was attended to at the hospital in Tubarão (SC) 1 h after the accident. **Figure 3A** shows a bee inside the ocular conjunctiva of the participant, demonstrating the aggressiveness of this accident. **Figure 3B** shows an Africanized honeybee.

### Clinical Outcomes

#### Adverse Events Not Related to the Investigational Product

Participant 103 developed an abscess on the lower-left flank (hypogastric region); participant 105 had inflammation of the right eye; participant 115 had strong-smelling urine, and pain in the testicular and lumbar regions (kidney); participant 113 had tachycardia and edema in the lower limbs (chronic hypertensive patient), and participant 111 had bronchospasms.

At follow-up visits, especially 10 d after treatment, all four participants, with moderate to severe cases, complained of intense itching in the lesions. This complaint was also mentioned by one of the participants who suffered a mild accident.

#### Adverse Events Related to the Investigational Product

During the AAV infusion, participant 110 presented with numb lips and itchy head; and participant 117 had pruritus and urticarial reactions. All AEs related to the product were adequately treated, and AAV infusion was completed. All adverse events, whether related or unrelated to the study, are described in **Data Sheet 3** - Clinical aspects.

### Clinical and Laboratory Outcomes

According to **Table 1 from Data Sheet 4 Tables-1-21**, from a clinical perspective, only one patient had hemodynamic alterations, whereas two presented with respiratory disorders. From a laboratory perspective, 15 had elevated CK levels, nine showed increased CRP levels, eight had leukocytosis, seven showed increased fibrinogen levels, and four showed increased ALT levels. All laboratory findings for CK, CRP, ALT, and complete blood count, including platelets count, fibrinogen, and creatinine levels, are available in the **Supplementary Materials in Data Sheet 5** -Laboratory exams.

The AHB scores assigned to the participants before the administration of AVV were 7, 6, and 5 or below 5, for 2, 5, and 13 participants, respectively (**Table 2, Data Sheet 4 -table 1-21**). The participant with the most severe case had a score of 7, and the 3 participants whose cases were considered to be of moderate severity had scores of 7 and 6, for 1 and 2 of them, respectively. All 16 participants whose cases were considered to be of mild severity had a score of 5 or less.

Laboratory tests showed an increase in CK, CRP, leukocytosis, fibrinogen, and ALT levels in 15, 9, 8, 7, and 4 participants, respectively. The biological AHB score, excluding clinical parameters, assigned to participants before the administration of AVV, was 5 in one, 4 in two, 3 in six, and score 2 or 1 in 11 participants respectively (**Table 6**, **Data Sheet 4 -Tables-1-21**).

As shown in **Table 18 of the Data Sheet 4 -Tables-1-21**, none of the participants showed clinical alterations 30 d after the administration of AAV. However, CRP, fibrinogen, CK, and ALT and leukocytosis, were altered in 4, 3, 2, and 1 of them, respectively.

As shown in **Table 21 of the Data Sheet 4 -Tables-1-21**, eight participants still had changes in the laboratory AHB score 30 d after AAV administration, with 1, 2, and 5 participants at scores 3, 2, and 1, respectively. Four participants, including 3 considered to have moderate cases and 1 considered to have a severe case, presented a laboratory AHB score of zero. Finally, **Table 5** shows the comparison between AHB scores before and 30 d after AAV treatment.

**Table 5:** Comparison between AHB scores before and 30 days after AAV treatment.

**Number of participants/AHB Scores**	**101**	**102**	**103**	**105**	**106**	**107**	**108**	**109**	**110**	**111**	**112**	**113**	**114**	**115**	**116**	**117**	**301**	**302**	**303**	**304**
AHB Scores before AAV treatment (*)	6	3	2	2	7	5	7	6	4	6	3	4	6	5	5	7	4	6	5	4
AHB Scores before AAV treatment (**)	3	1	1	1	5	3	4	4	2	2	1	2	3	2	3	3	2	3	2	2
AHB Scores 30 d after AAV treatment (***)	0	0	2	1	1	1	0	0	0	0	0	0	0	0	0	0	1	3	1	2

Colors: White – mild cases; Yellow – moderate cases; orange – severe cases.

(*) AHB Scores before AAV treatment, including clinical parameters.

(**) AHB Scores before AAV treatment, excluding clinical parameters.

(***) AHB scores 30 d after AAV treatment, excluding clinical parameters.

### Melittin and Phospholipase A_2_ Pharmacokinetics—ELISA Assays

All participants were monitored at 0, 2, 6, 12, 24, and 48 h of hospitalization, and at 10, 20, and 30 d of outpatient follow-up. Of the 20 participants, 6 had missed at least one of the scheduled sample collection sessions, and the ELISA test was not performed for 2 participants (301 and 303).

Fourteen participants had a complete follow-up, and all their blood samples for the different collection periods were obtained. Concentrations of melittin + phospholipase A_2_ varied between 0.03 ng/mL and 587.35 ng/mL during the hospitalization and follow-up period; despite the excellent clinical states of all the participants, melittin + phospholipase A_2_ concentrations ranged between 0 and 1.479 ng/mL.

**Figure 4** shows the time-course of melittin + phospholipase A_2_ levels in eighteen participants at admission, 2, 6, 12, 24, and 48 h after admission, and at 10, 20, and 30 d during the outpatient follow-up period. It is possible to observe that the blood concentration of melittin and PLA_2_ rises again especially after 10 days of admission, but without any clinical symptoms.

All the results of the ELISA assays are available in **Data Sheet 6**-Elisa assays.

### Mass Spectrometry

Analyses of the TIC chromatograms (**Figures 5A, B** **)** indicated that the 712.15 ion [M+4H^4+^], as well as the MS^2^ profiles, particularly that of the [y_13_]^2+^ fragment, made it possible to determine the presence and relative levels of melittin in the participants, as shown in **Table 6**. The relative quantity of melittin in the serum was determined based on the graphical interpretation of the spectra, as presented in the **Supplementary Materials**, in **Data Sheet 2** –**Figures-1**–**5**.

**Table 6:** Qualitative melittin detection in 19 participants 30 days after the AAV treatment.

No. of participants	00101	00102	00103	00105	00106	00107	00108	00109	00110	00111
Estimated number of stings	400	40	10	16	10	150	500	55	165	10
Number of antivenom vials	6	2	2	2	2	2	6	2	2	2
Presence of melittin	(+)	-	-	-	(+)	-	(*)	(+)	-	(++)
**No. of participants**	**00112**	**00113**	**00114**	**00115**	**00116**	**00117**	**00301**	**00302**	**00303**	**00304**
Estimated number of stings	30	50	500	100	180	2.000	20	150	7	50
Number of antivenom vials	2	2	6	2	2	10	2	2	2	2
Presence of melittin	-	-	(+)	(+++)	-	(+)	-	-	-	(++)

* (not evaluated) Colors: White – mild cases; Yellow – moderate cases; orange – severe cases.

## Discussion

Antivenom therapy was discovered in 1894 in France by Césaire Auguste Phisalix and Gabriel Bertrand (36), and Albert Calmette (37, 38). In Brazil, the discovery of this therapy had a profound impact on the work of Vital Brazil Mineiro da Campanha, a researcher has known worldwide for his scientific discoveries and for providing evidence of the specificity of antivenoms (39–41).

Since the 2000s, CEVAP has initiated different strategies to develop new antivenoms and therapies named “next antivenom approach”, including the development of a new Apilic antivenom (AAV), based on the association between a robust clinical protocol and an antivenom that strategically neutralizes only toxic molecules, i.e., melittin and phospholipase A2 (21). Then, allergens and nociceptive components were removed from the crude venom, significantly reducing suffering in the serum-producing animals; this directed the immune response of the herd toward the actual toxic and harmful compounds in envenomated humans. After the validation of the drug candidate, researchers from two Brazilian antivenom producers, the *Vital Brazil Institute (VBI)* and the *Butantan Institute (BI)*, joined the CEVAP team to produce a new AAV for clinical and pre-clinical trials.

In 2017, the Brazilian National Health Surveillance Agency (ANVISA), through Resolution RDC N ° 187 of November 8, 2017, established the minimum requirements for the registration of hyperimmune sera, with the aim of guaranteeing the quality, safety, and efficacy of these products. It also established that the request for the registration of these immunobiological must include the results of clinical trial studies. This is because, until then, Brazilian antivenoms were not validated through clinical trials (42). Therefore, this phase I/II clinical trial on the use of the AAV for the treatment of massive Africanized honeybee (*A. mellifera*) attacks, the clinical protocol of which was published in 2017 by Barbosa et al. (22), is the first to follow this new guideline.

The certainty diagnosis of envenoming by Africanized honey bees was made by the signs, symptoms, and by the clinical history of the patients, such as walking near an apiary, beekeepers handling swarms without the personal protective equipment (PPE), and, accidentally, when the people walked or played sports on the field (3, 5, 6). Second, according to the patient’s behavior in relation to the swarm because these bees attack victims and chase them for up to 400 meters (43, 44), moving at an average height of 1.62 m. Therefore, the head, trunk, and upper limbs are the parts of the human body most affected (13). Third, due to the presence of the stinger at the bee sting site - the only Hymenoptera in the world that dies after the bee sting (3, 45). Finally, due to the presence of melittin in the patients’ blood, evaluated by the Elisa assay and mass spectrometry - melittin is a peptide found only in the *Apis* genus (46). The main differential diagnosis was made with envenoming by social wasps common in Brazil (*Polybia* spp, *Polistes* spp, and *Solenopsis* spp). In this case, there would be no massive attack, no stinger at the bite site, and no melittin in the patient blood (10, 47).

According to WHO guidelines (48), clinical trials on antivenoms are designed to address three main issues: (a) the optimal initial dose; (b) efficacy, i.e., the ability of the antivenom to control the main clinical manifestations of envenoming; and (c) safety, i.e., the incidence and severity of early and late adverse reactions. Dose-finding studies are usually followed by randomized, controlled trials in which the new antivenom is compared to another antivenom already used, or in its absence, two doses of the new antivenom are compared (49).

As AAV was designed to specifically neutralize the two main toxic compounds of the venom, the clinical effects of low molecular mass proteins and peptides, that cause manifestation such as intense pruritus, flushing, hyperthermia, papules, urticarial plaques, hypotension, tachycardia, headache, nausea, vomiting, abdominal colic, bronchospasms, and psychomotor agitation, were neutralized by symptomatic medications such as antihistamines, corticosteroids, adrenaline (when the anaphylactic shock was suspected), and pethidine hydrochloride (for severe pain). In case of the occurrence of bronchospasms, oxygen (O_2_) catheters associated with β-2-agonist-type inhaled bronchodilators (salbutamol, fenoterol, or terbutaline) were used at the usual recommended doses (22). As the main objective of this study was to assess the safety of AAV, including the severity of acute adverse events, and to confirm its lowest effective dose when confronted with different amounts of inoculated venom, the main adverse events caused by equine-heterologous antivenom therapy were studied.

Safety was assessed mainly through early adverse events, particularly, IgE-mediated anaphylactic reactions type I, non-IgE-mediated anaphylactic reactions, pyrogenic reactions (endotoxin contamination), and late adverse reactions (type III hypersensitivity, such as serum sickness) (50–53).

Regarding adverse events unrelated to the product, the persistence of itching the sting sites at the first follow-up outpatient, i.e., 10 d after AAV administration, was highlighted. This clinical manifestation and its persistence at the sting site or in the subcutaneous cellular tissue are related to envenoming (4, 6, 7, 18). Since the clinical protocol recommended the use of antihistamines only during the hospitalization period, this point should be revised and the use of this medication should be extended to at least 15 days after discharge.

Regarding the adverse events related to this product, only two participants (10%) experienced mild and early adverse reactions, such as numbness of the lips, itchy head, pruritus, and urticarial reactions. Recently, Mendonça-da-Silva et al. (54) evaluated the safety and efficacy of a freeze-dried trivalent antivenom for snakebites in the Brazilian Amazon, with 112 participants treated in an open-label, randomized controlled phase IIb clinical trial. Twenty-three (19.8%) participants experienced early adverse events after antivenom therapy. The most common symptoms were urticaria (13.8%), pruritus (11.2%), facial flushing (3.4%), and vomiting (3.4%). Our results are similar to those of Mendonça-da-Silva et al. (54), and those of other studies in the literature (50–53). None of the participants developed serum sickness. AAV appears to be a safe investigational product for clinical use.

It should be emphasized that in the event of an accident involving AHB, classically described adverse reactions due to antivenoms may be confused with adverse events of the envenoming itself. Therefore, it becomes difficult to determine whether they are caused by the venom or AAV (4, 6, 7, 18). Despite these limitations, it was possible to conclude that adverse events related to the investigational product were similar to those caused by common antivenoms used in other types of envenomings (45–49); thus, confirming the safety of AAV for clinical use.

As concerns clinical and laboratory outcomes, the authors proposed a new AHB score to assess the severity of the participants’ cases at the time of the first visit, in addition to the evolutive outcome. The use of the AHB score proved to be simple and showed proportionality to the severity of the envenoming, and the normalization of clinical and biological signs. As it combines clinical and biological criteria, the score appears to be both sensitive and accurate, particularly with respect to biological variables, for the characterization of the severity and course of envenoming. This allowed us to confirm clinical remission in all participants 30 d after AAV administration. However, biological alterations persisted in some participants (8/20) even though they did not present with particularly severe envenomings. Surprisingly, the two participants with the most severe cases of envenoming, who received 500 and 2,000 stings, and were assigned an AHB score of 7, had a score of zero 30 d after AAV administration. In addition, all the 5 participants who had an AHB score of 6 at inclusion, had a score of zero 30 d after discharge; but one remained at 1 because of a CRP level slightly above the limit (1.1 mg/dL). It should also be emphasized that envenoming caused by venomous animals triggers a systemic inflammatory response syndrome described by several authors since the 1990s (55–59). This constitutes an acute phase reaction with a massive release of pro-inflammatory cytokines (particularly, IL-1, IL-6, and TNF alpha), and acute-phase proteins, especially CRP. These acute-phase reactions are in accordance with previous clinical studies performed on other antivenoms (4, 6, 55–60).

ELISA tests, standardized to assess the presence of melittin and phospholipase A_2_ in this study, showed the reappearance of these two fractions 10, 20, and 30 d after treatment in most participants.

In a study using an ovine model to evaluate the interactions between the venom produced by *Micrurus fulvius* and a F(ab’)_2_ antivenom administered intravenously, Paniagua et al. (61) observed an immediate neutralization of the venom in the bloodstream. Antivenom appears not to be able to neutralize the residual venom deposited at the site of the sting; so, the venom can remain active, with slow-release into the bloodstream and consequently be distributed systemically (61). However, as long as the antivenom remains in the plasma compartment, the venom is captured and eliminated until the plasma antivenom concentration is no longer sufficient to neutralize the released venom (62–64). This phenomenon is well illustrated by the use of Fab, which has a short half-life of approximately 10-15 h (62), in the treatment of envenoming caused by Crotalidae; this treatment requires constant re-administration of the Fab antivenom (65, 66). Under these conditions, the renewal of the administration of the antivenom serum must be planned based on the half-life of the F(ab’)_2_ antibodies, which is approximately 50 h (62), and the quantity of bee venom in circulation, which is more difficult to determine.

We hypothesize that, in consonance with the findings of Paniagua et al. (61), multiple bee stings occur in many parts of the human body, depositing the venom in the subcutaneous tissue from where it is slowly released to the bloodstream. In addition to the clearance of the F(ab’)_2_ molecule, the latter remains in the plasma compartment without entering the tissues, preventing the neutralization of the venom in the subcutaneous cellular tissue (62). Surprisingly, participants who received only two vials of AAV were those who still presented with a systemic inflammatory response syndrome 30 d after treatment, i.e., an inflammatory response based on increased CRP levels (4 participants), fibrinogen levels (3 participants), and leukocyte count (1 participant) (4, 54–59). This can be explained by a lower F(ab’)_2_ concentration, which is eliminated more quickly, permitting the venom, even in small quantities, to stay longer in the body. This clearly raises the question of renewing AAV administration in participants who suffered multiple AHB stings a week after the first dose or increasing the number of vials at the first administration, even if the initial symptoms were mild.

Finally, qualitative mass spectrometry (8) showed the presence of melittin in the blood of participants with mild (five), moderate (two), and severe (one) cases 30 d after AAV administration. It should be emphasized that participant 115, who received 2 vials of AAV after being stung by approximately 100 honeybees, experienced a particular increase in circulating melittin 30 d after treatment. Thus, the clinical response of the participants, laboratory test results from the acute-phase response, ELISA tests, and mass spectrometry allowed us to infer that although the investigational product is safe (which was the main objective of this study), it would be necessary to revise the clinical protocol, especially concerning the number of vials of AAV to be administered. A multicenter phase III clinical trial should be performed to confirm these hypotheses and adjust the doses of this new antivenom.

## Conclusions

AAV proved to be safe, as related adverse events were observed in only two (10%) participants, corroborating reports on heterologous antivenom use. No late adverse events were observed on d 10, 20, or 30 during clinical surveillance. Preliminary efficacy was observed by clinical improvement in participants, a decrease in acute-phase-reaction markers, and a reduction in circulating melittin and phospholipase A_2_ levels, measured using ELISA. The doses recommended in the clinical protocol should be reassessed and increased, given that melittin was founding the blood of eight participants 30 d after the specific treatment, through mass spectrometry. It should be noted that these issues were expected, as accidents involving AHB are peculiar and different from all other venomous animal accidents described. A phase III clinical trial should be performed to confirm these observations, adjust the recommended doses, and assess the efficacy of the product.

## Data Availability Statement

The raw data supporting the conclusions of this article will be made available by the authors, without undue reservation.

## Ethics Statement

The clinical protocol has been previously approved by the Brazilian National Commission on Ethics in Research (CONEP, Certificate of Presentation of Ethical Appreciation No. 19006813.4.1001.5411, v7, approved in 06/07/2016) and the Brazilian National Health Surveillance Agency (ANVISA) whose Consent Record of Apis Study was approved on 02/05 2016 by No. 0907532142, Proc. No. 25361611582201493. This trial RBR-3FTHF8 was registered in 2015 in the Brazilian Clinical Trials Registry (ReBEC) (24). The first participant was included in 08/22/2016, the Universal Trial Number (UTN) is U1111-1160-7011, the Register Number is RBR-3fthf8 and the public access URL is available at http://www.ensaiosclinicos.gov.br/rg/RBR-3fthf8/. The patients/participants provided their written informed consent to participate in this study. Written informed consent was obtained from the individual(s) for the publication of any potentially identifiable images or data included in this article.

## Author Contributions

AB, FS-T, and MM were the principal investigators. FC, BM, and LS standardized the ELISA assay. RF and LC produced and performed quality control on the Apilic Antivenom. JB, DT, NM, CC, MC, and AO recruited the participants. DP developed the mass spectrometry assay. RF, LB, J-PC, and BB discussed the proposal and corrected the manuscript. All authors contributed to the article and approved the submitted version.

## Funding

This study was supported by the National Council of Technological and Scientific Development (CNPq) under Grant No. 437089/2018-5 (LS), 563582/2010-3 (BB), and 401170/2013-6 (BB), and in part by grants from the Coordination for the Improvement of Higher Education Personnel (CAPES) [AUX-PE Toxinology Proc. No. 23,038.000823/201121]. RF is a CNPq PQ1C fellow researcher [303224/2018-5]. DP is a CNPq fellow researcher [301974/2019-5]. BB is a CNPq PQ-2 fellow researcher [306339/2020-0]. FAPESP Grant No. 2020/09819-6 and No. 2020/16747-1 (BM, LS, RF). PROPG/UNESP Edital 01/2021. DECIT/MS No. 841906/2016-SICONV and No.841904/2016- SICONV (RF).

## Conflict of Interest

RF is a CNPq PQ1C fellow researcher, process number 303224/2018-5.

RF, BB, LC, and DP participated as authors in patent application BR10201502630. Deposit: 10/16/2015. PCT deposit number: 10201502630. PCT deposit date: 10/16/2015 “Process of obtaining anti-apilic equine serum and its uses” INPI: National Institute of Industrial Property, Brazil.

The remaining authors declare that the research was conducted in the absence of any commercial or financial relationships that could be construed as a potential conflict of interest.

## References

[1] Scott Schneider S DeGrandi-Hoffman G Smith DR. The African honey bee: factors contributing to a successful biological invasion. Annu Rev Entomol (2004) 49:351–76. 10.1146/annurev.ento.49.061802.123359 14651468

[2] Oliveira GP Kadri SM Benaglia BGE Ribolla PEM Orsi RO. Energetic supplementation for maintenance or development of *Apis mellifera* L. colonies. J Venom Anim Toxins incl Trop Dis (2020) 26:e20200004. 10.1590/1678-9199-jvatitd-2020-0004 32518557 PMC7250130

[3] Ferreira RS Jr Almeida R Barraviera SRS Barraviera B. Historical perspective and human consequences of Africanized bee stings in the Americas. J Toxicol Environ Health Part B (2012) 15:97–108. 10.1080/10937404.2012.645141 22401177

[4] United States Department of Agriculture Agricultural Research Service. ARS Honey Bee Health (2021). Available at: https://www.ars.usda.gov/oc/br/ccd/index/ (Accessed January 7, 2021).

[5] Pucca MB Cerni FA Oliveira IS Jenkins TP Argemí L Sørensen CV. Bee updated: Current knowledge on bee venom and bee envenoming therapy. Front Immunol (2019) 10:2090. 10.3389/fimmu.2019.02090 31552038 PMC6743376

[6] Almeida R Olivo T Mendes RP Barraviera SRCS Souza LR Martins JG. Africanized honeybee stings: How to treat them. Rev Soc Bras Med Trop (2011) 44(6):755–61. 10.1590/S0037-86822011000600020 22231250

[7] Ownby CL Powell JR Jiang M Fletcher JE. Melittin and phospholipase A2 from bee (*Apis mellifera*) venom cause necrosis of murine skeletal muscle *in vivo* . Toxicon (1997) 35(1):67–80. 10.1016/s0041-0101(96)00078-5 9028010

[8] Ferreira RS Jr Sciani JM Marques-Porto R Junior AL Orsi RO Barraviera B. Africanized honey bee (*Apis mellifera*) venom profiling: Seasonal variation of melittin and phospholipase A2 levels. Toxicon (2010) 56(3):355–62. 10.1016/j.toxicon.2010.03.023 20403370

[9] Sciani JM Marques-Porto R Lourenço A Orsi RO Ferreira RS Jr Barraviera B. Identification of a novel melittin isoform from Africanized *Apis mellifera* venom. Peptides (2010) 31(8):1473–9. 10.1016/j.peptides.2010.05.001 20472009

[10] Santos LD Pieroni M Menegasso ARS Pinto JRAS Palma MS. A new scenario of bioprospecting of Hymenoptera venoms through proteomic approach. J Venom Anim Toxins incl Trop Dis (2011) 17(4):364–77. 10.1590/S1678-91992011000400003

[11] World Health Organization (WHO). Report of the tenth meeting of the WHO: Strategic and Technical Advisory Group for Neglected Tropical Diseases (2017). Geneva: WHO. Available at: http://www.who.int/neglected_diseases/NTD_STAG_report_2017.pdf?ua=1 (Accessed January 7, 2021).

[12] Chippaux JP. Snakebite envenomation turns again into a neglected tropical disease! J Venom Anim Toxins incl Trop Dis (2017) 23:38. 10.1186/s40409-017-0127-6 28804495 PMC5549382

[13] Oliveira SK Trevisol DJ Parma GC Ferreira RS Jr Barbosa AN Barraviera B. Honey bee envenoming in Santa Catarina, Brazil, 2007 through 2017: an observational, retrospective cohort study. Rev Soc Bras Med Trop (2019) 52:e20180418. 10.1590/0037-8682-0418-2018 30994807

[14] BRAzIL – Ministry of Health (MS). Epidemiological situation - Health Information (TABNET), Accidents by Venomous Animals, Bee (2020). Available at: http://www2.datasus.gov.br/DATASUS/index.php?area=0203&id=29878153 (Accessed January 7, 2021).

[15] Coombs RRA Gell PGH. “Classification of allergic reactions responsible for clinical hypersensitivity and disease”. In: Gell PHG Coombs RRA, editors. Clinical aspects of immunology, 2nd edition. Oxford and Edinburgh: Blackwell Scientific Publ. (1968). p. 575–96.

[16] Caldas SA Graça FA Barros JS Rolim MF Peixoto TC Peixoto PV. Lesions caused by Africanized honeybee stings in three cattle in Brazil. J Venom Anim Toxins incl Trop Dis (2013) 19:18. 10.1186/1678-9199-19-18 23968247 PMC3765377

[17] Silva GBD Jr Vasconcelos AG Jr Rocha AMT Vasconcelos VR Barros JN Fujishima JS. Acute kidney injury complicating bee stings - a review. Rev Inst Med Trop Sao Paulo (2017) 59:e25. 10.1590/S1678-9946201759025 28591253 PMC5459532

[18] França FO Benvenuti LA Fan HW Santos DR Hain SH Picchi-Martins FR. Severe and fatal mass attacks by ‘killer’ bees (Africanized honey bees - Apis mellifera scutellata) in Brazil: clinicopathological studies with measurement of serum venom concentrations. Q J Med (1994) 87(5):269–82. 10.1093/oxfordjournals.qjmed.a068927 7938407

[19] Santos KS Stephano MA Marcelino JR Ferreira VM Rocha T Caricati C. Production of the first effective hyperimmune equine serum antivenom against Africanized bees. PLoS One (2013) 8(11):e79971. 10.1371/journal.pone.0079971 24236166 PMC3827448

[20] Ferreira RS Jr Anderlini RP Sant’Anna OABE Pimenta DC Orsi RO Barraviera B. New nanostructured silica adjuvant (SBA-15) employed to produce antivenom in young sheep using *Crotalus durissus terrificus* and *Apis mellifera* venoms detoxified by cobalt-60. J Toxicol Environ Health Part A (2010) 73(13-14):926–33. 10.1080/15287391003745069 20563926

[21] Africanized Honeybee Antivenom. Movie (2015). Available at: https://youtu.be/y2cvGH7X6D8 (Accessed January 7, 2021).

[22] Barbosa AN Boyer L Chippaux JP Medolago NB Caramori CA Paixão AG. A clinical trial protocol to treat massive Africanized honeybee (*Apis mellifera*) attack with a new apilic antivenom. J Venom Anim Toxins incl Trop Dis (2017) 23:14. 10.1186/s40409-017-0106-y 28331487 PMC5356296

[23] Teixeira-Cruz JM Strauch MA Monteiro-Machado M Tavares-Henriques MS Moraes JA Cunha LER. A novel apilic antivenom to treat massive, Africanized honeybee attacks: a preclinical study from the lethality to some biochemical and pharmacological activities neutralization. Toxins (2021) 13(1):30. 10.3390/toxins13010030 33466223 PMC7824798

[24] Brazilian Clinical Trials Registry (ReBEC). Available at: http://www.ensaiosclinicos.gov.br/rg/RBR-3fthf8/ (Accessed January 7, 2021).

[25] Knaus WA Draper EA Wagner DP Zimmerman JE. APACHE II: a severity of disease classification system. Crit Care Med (1985) 13(10):818–29. 10.1097/00003246-198510000-00009 3928249

[26] Madden AM Smith S. Body composition and morphological assessment of nutritional status in adults: a review of anthropometric variables. J Hum Nutr Diet (2016) 29(1):7–25. 10.1111/jhn.12278 25420774

[27] McPherson RA Pincus MR. Henry"s Clinical Diagnosis and Management by Laboratory Methods. USA: Elsevier Missouri (2017). p. 1584, ISBN: 978-0-323-29568-0.

[28] Kidney Disease: Improving Global Outcomes (KDIGO) CKD Work Group. KDIGO 2017 clinical practice guideline update for the diagnosis, evaluation, prevention, and treatment of Chronic Kidney Disease - Mineral and Bone Disorder (CKD-MBD). Kidney Int Suppl (2017) 7(1):1–59. 10.1016/j.kisu.2017.04.001 PMC6340919 30675420

[29] Brown SGA. Clinical features and severity grading of anaphylaxis. J Allergy Clin Immunol (2004) 114:371–6. 10.1016/j.jaci.2004.04.029 15316518

[30] Silva HA Pathmeswaran A Ranasinha CD Jayamanne S Samarakoon SB Hittharage A. Low-dose adrenaline, promethazine, and hydrocortisone in the prevention of acute adverse reactions to antivenom following snakebite: a randomized, double-blind, placebo-controlled trial. PloS Med (2011) 8(5):e1000435. 10.1371/journal.pmed.1000435 21572992 PMC3091849

[31] Chávez-Olórtegui C Fonseca SCG Campolina D Amaral CFS Diniz CR. ELISA for the detection of toxic antigens in experimental and clinical envenoming by *Tityus serrulatus* scorpion venom. Toxicon (1994) 32(12):1649–56. 10.1016/0041-0101(94)90323-9 7725332

[32] Nakane PK Kawoi A. Peroxidase-labeled antibody. A new method of conjugation. J Histochem Cytochem (1974) 22(12):1084–91. 10.1177/22.12.1084 4443551

[33] Bucaretchi F Mello SM Vieira RJ Mamoni RL Blotta MHSL Antunes E. Systemic envenomation caused by the wandering spider *Phoneutria nigriventer*, with quantification of circulating venom. Clin Toxicol (2008) 46(9):885–9. 10.1080/15563650802258524 18788004

[34] Hackshaw A. A concise guide to clinical trials. Oxford: Wiley-Blackwell (2009). p. 224, ISBN: 978-1-405-16774-1.

[35] Zar JH. Bioestatistical analysis. New Jersey: Prentice Hall (2010). p. 944, ISBN: 978-8-178-08965-2.

[36] Bochner R. Paths to the discovery of antivenom serotherapy in France. J Venom Anim Toxins incl Trop Dis (2016) 22:20. 10.1186/s40409-016-0074-7 27279829 PMC4898362

[37] Phisalix C Bertrand G. Sur la propriété antitoxique du sang des animaux vaccinés contre le venin de vipère. C R Soc Biol (1894) 46:111–3.

[38] Calmette A. L’immunisation artificielle des animaux contre le venin des serpents, et la thérapeutique expérimentale des morsures venimeuses. C R Soc Biol (1894) 46:120–4.

[39] Lucas SM. The history of venomous spider identification, venom extraction methods and antivenom production: a long journey at the Butantan Institute, São Paulo, Brazil. J Venom Anim Toxins incl Trop Dis (2015) 21:21. 10.1186/s40409-015-0020-0 26085831 PMC4470033

[40] Pucca MB Cerni FA Janke R Bermúdez-Méndez E Ledsgaard L Barbosa JE History of envenoming therapy and current perspectives. Front Immunol (2019) 10:1598. 10.3389/fimmu.2019.01598 31354735 PMC6635583

[41] Grego KF Vieira SEM Vidueiros JP Serapicos EO Barbarini CC da Silveira GPM Maintenance of venomous snakes in captivity for venom production at Butantan Institute from 1908 to the present: a scoping history. J Venom Anim Toxins Incl Trop Dis (2021) 27:e20200068. 10.1590/1678-9199-JVATITD-2020-0068 PMC7856910 33597972

[42] BRAZIL, Ministry of Health, National Health Surveillance Agency. Resolução - RDC N° 187 (2017). Available at: https://www.in.gov.br/materia/-/asset_publisher/Kujrw0TZC2Mb/content/id/19402576/do1-2017-11-09-resolucao-rdc-n-187-de-8-de-novembro-de-2017-19402357#:~:text=1%C2%BA%20Estabelece%20os%20requisitos%20m%C3%ADnimos,seguran%C3%A7a%20e%20aefic%C3%A1cia%20destes%20produtos.&text=Abrang%C3%AAncia-,Art.,fins%20de%20concess%C3%A3o%20de%20registro (Accessed January 7, 2021).

[43] Collins AM Rinderer TE Tucker KW. Colony defence of two honeybee types and their hybrid 1. Naturally mated queens. J Apic Res (1988) 27(3):137–40. 10.1080/00218839.1988.11100793

[44] Silveira DC Maracajá PB Silva RA Sousa RM Sotoblanco B. Diurnal and seasonal changes of defensive behavior of Africanized bees (“*Apis mellifera*” L.). Rev Bras Saúde Prod Anim (2015) 16(4):925–34. 10.1590/S1519-99402015000400016

[45] Fitzgerald KT Flood AA. Hymenoptera stings. Clin Tech Small Anim Pract (2006) 21(4):194–204. 10.1053/j.ctsap.2006.10.002 17265905

[46] Moreno M Giralt E. Three valuable peptides from bee and wasp venoms for therapeutic and biotechnological use: melittin, apamin and mastoparan. Toxins (2015) 7(4):1126–50. 10.3390/toxins7041126 PMC4417959 25835385

[47] Souza MM Teofilo-Guedes GS Bueno ET Milani LR Souza ASB. Social wasps (Hymenoptera, Polistinae) from the Brazilian savanna – review. Sociobiology (2020) 67(2):129–38. 10.13102/sociobiology.v67i2.4958

[48] World Health Organization. WHO Guidelines for the Production, Control and Regulation of Snake Antivenom Immunoglobulins. Geneva, Switzerland: World Health Organization (2018). Available at: http://www.who.int/bloodproducts/snake_antivenoms/snakeantivenomguide/en/ (Accessed January 7, 2021).

[49] Gutiérrez JM Solano G Pla D Herrera M Segura Á Vargas M. Preclinical evaluation of the efficacy of antivenoms for snakebite envenoming: State-of-the-art and challenges ahead. Toxins (2017) 9(5):163. 10.3390/toxins9050163 PMC5450711 28505100

[50] Morais V. Antivenom therapy: efficacy of premedication for the prevention of adverse reactions. J Venom Anim Toxins incl Trop Dis (2018) 24:7. 10.1186/s40409-018-0144-0 29507580 PMC5831611

[51] Gutiérrez JM. Global availability of antivenoms: The relevance of Public Manufacturing Laboratories. Toxins (2018) 11(1):5. 10.3390/toxins11010005 PMC6356591 30586868

[52] León G Herrera M Segura Á Villalta M Vargas M Pathogenic GJM. Mechanisms underlying adverse reactions induced by intravenous administration of snake antivenoms. Toxicon (2013) 76:63–76. 10.1016/j.toxicon.2013.09.010 24055551

[53] Silva HA Ryan NM de Silva HJ. Adverse reactions to snake antivenom, and their prevention and treatment. Br J Clin Pharmacol (2016) 81(3):446–52. 10.1111/bcp.12739 PMC4767202 26256124

[54] Mendonça-da-Silva I Magela-Tavares A Sachett J Sardinha JF Zaparolli L Gomes-Santos MF. Safety and efficacy of a freeze-dried trivalent antivenom for snakebites in the Brazilian Amazon: An open randomized controlled phase IIb clinical trial. PloS Negl Trop Dis (2017) 11(11):e0006068. 10.1371/journal.pntd.0006068 29176824 PMC5720814

[55] Lomonte B Tarkowski A Hanson LÅ. Host response to *Bothrops asper* snake venom: analysis of edema formation, inflammatory cells, and cytokine release in a mouse model. Inflammation (1993) 17:93–105. 10.1007/BF00916097 8491517

[56] Barraviera B Lomonte B Tarkowski A Hanson LÅ Meira DA. Acute-phase reactions, including cytokines, in patients bitten by *Bothrops* and *Crotalus* snakes in Brazil. J Venom Anim Toxins (1995) 1(1):11–22. 10.1590/S0104-79301995000100003

[57] Sofer S. Scorpion envenomation. Intensive Care Med (1995) 21:626–8. 10.1007/BF01711538 8522664

[58] Paolino G Di Nicola MR. Letter to the Editor: Acute-phase response fever in Viperidae as a potential and additional clinical sign. Toxicon (2020) 184:229–30. 10.1016/j.toxicon.2020.07.008 32681849

[59] Voronov E Apte RN Sofer S. The systemic inflammatory response syndrome related to the release of cytokines following severe envenomation. J Venom Anim Toxins (1999) 5(1):5–33. 10.1590/S0104-79301999000100002

[60] Slevin M Molins B. Editorial: C-Reactive protein in age-related disorders. Front Immunol (2018) 9:2745. 10.3389/fimmu.2018.02745 30524450 PMC6258772

[61] Paniagua D Vergara I Román R Romero C Benard-Valle M Calderón A. Antivenom effect on lymphatic absorption and pharmacokinetics of coral snake venom using a large animal model. Clin Toxicol (2019) 57(8):727–34. 10.1080/15563650.2018.1550199 30773936

[62] Rivière G Choumet V Audebert F Sabouraud A Debray M Scherrmann JM. Effect of antivenom on venom pharmacokinetics in experimentally envenomed rabbits: toward an optimization of antivenom therapy. J Pharmacol Exp Ther (1997) 281(1):1–8. 9103473

[63] Rivière G Choumet V Saliou B Debray M Bon C. Absorption and elimination of viper venom after antivenom administration. J Pharmacol Exp Ther (1998) 285(2):490–5. 9580588

[64] Barraviera B Sartori A Silva MFP Kaneno R Peraçoli MTS. Use of an ELISA assay to evaluate venom, antivenom, IgG and IgM human antibody levels in serum and cerebrospinal fluid from patients bitten by *Crotalus durissus terrificus* in Brazil. J Venom Anim Toxins (1996) 2(1):14–27. 10.1590/S0104-79301996000100003

[65] Boyer LV Seifert SA Clark RF McNally JT Williams SR Nordt SP. Recurrent and persistent coagulopathy following pit viper envenomation. Arch Intern Med (1999) 159:706–10. 10.1001/archinte.159.7.706 10218750

[66] Boyer LV Seifert SA Cain JS. Recurrence phenomena after immunoglobulin therapy for snake envenomations: Part 2. Guidelines for clinical management with crotaline Fab antivenom. Ann Emerg Med (2001) 37(2):196–201. 10.1067/mem.2001.113134 11174239

