# Supplementary material for: Single-Arm, Multicenter Phase I/II Clinical Trial for the Treatment of Envenomings by Massive Africanized Honey Bee Stings Using the Unique Apilic Antivenom
Source: Front Immunol. 2021 Mar 23;12:653151. doi: 10.3389/fimmu.2021.653151 (PMC8025786; doi:10.3389/fimmu.2021.653151)
Supplement: Supplementary file 1 [file DataSheet_1.docx]

**Data Sheet 1 - Supplemental material of procedures and protocols of laboratory exams collected**

**Table 1.** Procedures and laboratory exams evaluated during the periods of study.

| **Procedures and Laboratory exams** | **Before treatment** | **Discharge day** | **10 days after the treatment** | **20 days after the treatment** | **30 days after the treatment** |
| --- | --- | --- | --- | --- | --- |
| Signature of FICF | X | - | - | - | - |
| Personal data | X | - | - | - | - |
| Clinical history | X | X | X | X | X |
| Physical exam | X | X | X | X | X |
| Concomitant medications | X | X | X | X | X |
| Adverse events | X | X | X | X | X |
| Vital signs | X**^a^** | X | X | X | X |
| Hematocrit | X | X | X | X | X |
| Haemoglobin | X | X | X | X | X |
| Leukocytes | X | X | X | X | X |
| Platelets | X**^b^** | X | X | X | X |
| ESR | X | X | X | X | X |
| Creatinine | X | X | X | X | X |
| CRP | X | X | X | X | X |
| ALT | X | X | X | X | X |
| Fibrinogen | X**^b^** | X | X | X | X |
| CPK | X**^b^** | X | X | X | X |
| PT | X^b^ | X | X | X | X |
| APTT | X^b^ | X | X | X | X |
| ELISA assays | X^c^ | X | X | X | X |
| Pregnancy test | X^e^ | - | - | - | - |

FICF - Free and Informed Consent Form; ESR - erythrocyte sedimentation rate; CRP – C-reactive protein; ALT – alanine amino transferase; CPK – creatino fosfoquinase; PT - Prothrombin time; APTT - activated partial thromboplastin time

**Table 2.** Normal values of laboratory tests carried out in the two participating centers, Botucatu (SP) and Tubarão (SC) (27, 28).

| **Participating centers and laboratory exams** | **Botucatu** | **Tubarão** |
| --- | --- | --- |
| CPK | 55 to 170 U/L | 24 a 195 U/L |
| ALT | M < 72 U/L; F < 52 U/L | < 42 U/L |
| Creatinine | 0.80 to 1.50 mg/dL | 0.40 a 1.40 mg/dL |
| CRP | < 1 mg/dL | < 5 mg/L |
| Fibrinogen | 200 to 393 mg/dL | 200 to 400 mg/dL |
| Leucocytes | 4 to 11 x 10^3^/mm^3^ | 5 to 10 x 10^3^/mm^3^ |
| Platelets | 140 to 440 x 10^3^/mm^3^ | 140 to 400 x 10^3^/mm^3^ |

CPK = creatine phosphokinase, ALT = alanine amino transferase. CRP = C-reactive protein, M = male, F = female
