# Supplementary material for: Single-Arm, Multicenter Phase I/II Clinical Trial for the Treatment of Envenomings by Massive Africanized Honey Bee Stings Using the Unique Apilic Antivenom
Source: Front Immunol. 2021 Mar 23;12:653151. doi: 10.3389/fimmu.2021.653151 (PMC8025786; doi:10.3389/fimmu.2021.653151)
Supplement: Supplementary file 2 [file DataSheet_2.docx]

**Data Sheet 2 –** **Supplemental material of Mass spectrometry analyses**

**A**

**B**

**Figure 1. A) MS spectra of purified melittin. B) Zoomed spectrum showing the multiply charged melittin [M+4H^4+^]**

**A**

**B**

**Figure 2. A) MS^2^ fragmentation spectrum of melittin [M+4H^4+^]. B) Zoomed spectrum showing the multiply charged [y_13_]^2+^ fragment.**

**A**

**B**

**Figure 3. Participant 00101 MS (A) and MS^2^ (B) spectra, considered as ‘+’**

**A**

**B**

**Figure 4. Participant 00111 MS (A) and MS^2^ (B) spectra, considered as ‘++’.**

A

B

**Figure 5. Participant 00115 MS (A) and MS^2^ (B) spectra, considered as ‘+++’**
