# Supplementary material for: Single-Arm, Multicenter Phase I/II Clinical Trial for the Treatment of Envenomings by Massive Africanized Honey Bee Stings Using the Unique Apilic Antivenom
Source: Front Immunol. 2021 Mar 23;12:653151. doi: 10.3389/fimmu.2021.653151 (PMC8025786; doi:10.3389/fimmu.2021.653151)
Supplement: Supplementary file 3 [file DataSheet_3.docx]

**Data Sheet 3 – Supplemental material of Clinical aspects**

**00101 – Participant classified as a moderate case**

**The time elapsed between the accident and infusion of apilic antivenom was three days.**

- Participant spontaneously presented after hospital discharge and before the first return visit, which would be 10 days after, with a complaint of bilateral swelling in the lower limbs, bilateral pain in the trapezius muscle, pain in the right knee, and severe itching in the lesions. The patient denied fever.
- The patient was medicated with: Caffeine 30 mg + Carisoprodol 125 mg + Diclofenac sodium 50 mg + Paracetamol 300 mg
- ***Adverse events*:** an increase in levels of leukocytes during hospitalization.

**Participant 00102**

**The time elapsed between the accident and infusion of apilic antivenom was 10 days.**

- On the second return visit, that is, 20 days after hospital discharge, he reported recurrent generalized muscle pain, having used Dorflex, and referenced bilateral lower limb swelling, bilateral pain in the trapezius muscle, and pain in the right knee. He denied fever.
- He was medicated with: Caffeine 30 mg + Carisoprodol 125 mg + Diclofenaco sodium 50 mg + Paracetamol 300 mg
- ***Adverse events*:** generalized muscle pain.

**Participant 00103**

**The time elapsed between the accident and infusion of apilic antivenom was less than 24 hours**

- Patient reported having rheumatoid arthritis
- At the first return visit, that is, 10 days after the serotherapy, the patient reported arthralgia in a right-hand finger initiated after a bee sting on this finger.
- Appeared at Upeclin (Clinical Research Unit – Botucatu Medical School) between the R2 and R3 returns, that is, 20 days after the accident reporting having noticed, three days prior, the presence of a nodule in the abdominal region.
- Upon physical examination, the patient presented an abscess of 10 cm on the lower-left flank in the hypogastric region, with a drainage point and purulent secretion.
- The following treatment was initiated: Clavulanate + amoxicillin for 10 days. Nimesulide treatment for arthralgia was continued.
- ***Adverse events*:** abscess on the lower-left flank in the hypogastric region, hyperthermia possibly related to the abscess, worsening of rheumatoid arthritis, as well as referencing occasional dizziness and nausea. Maintenance of elevated levels of C-reactive protein and CPK 30 days after specific treatment

**Participant 00105**

**The time elapsed between the accident and infusion of apilic antivenom was less than 24 hours**

- Reported on the third return visit, that is, 30 days after the treatment, inflammation in the right eye in addition to generalized itching of the body.
- Was medicated with: Maxitrol eye drops (Neomycin + Dexamethasone). Olopatadine for 30 days
- ***Adverse events*:** ocular inflammation. Maintenance of elevated ALT levels 30 days after the specific treatment

**Participant 00106**

**The time elapsed between the accident and infusion of apilic antivenom was two days.**

- ***Adverse events***: maintenance of elevated levels of C-reactive protein 30 days after the specific treatment

**Participant 00107**

- **The time elapsed between the accident and infusion of apilic antivenom was one day.**
- Patient reported having hypothyroidism and arterial hypertension
- Presented before antivenom infusion: hyperthermia and arterial hypertension
- Medicated with: Losartan, Atenolol, and Levothyroxine. At hospital discharge, Rosuvastatin and Clonidine were indicated.
- On the first return visit, that is, 10 days after serotherapy, arterial pressure showed a tendency toward normalization.
- ***Adverse events****:* CPK was elevated at hospital discharge and maintained until the first return, that is, 10 days after the specific treatment. High fibrinogen levels were maintained 30 days after the specific treatment

**00108 – Participant classified as a moderate case**

- **The time elapsed between the accident and infusion of apilic antivenom was 19 days.**
- At hospital discharge Amoxicillin + Clavulanate were indicated for infection at sting sites.
- There was a loss of follow-up starting at the first return.

**Participant 00109**

- **The time elapsed between the accident and infusion of apilic antivenom was two days.**
- At the final return visit, that is, 30 days after treatment presented two erythematous maculopapular lesions in the left cubital fossa.
- Medicated with: dexamethasone for 5 days; was considered an unrelated event.
- ***Adverse events:*** increase of CPK, creatinine, CRP, and leukocytosis before the specific treatment.

**Participant 00110**

- **The time elapsed between the accident and infusion of apilic antivenom was two days.**
- Patient reports being a chronic alcohol abuser, in addition to having chronic hepatitis B and megaloblastic anemia.
- Patient reports as an antecedent, having had anaphylactic shock triggered by a bee sting about 6 years prior.
- At 30 minutes after initiating antivenom infusion, the patient presented sensation of numb lips, itchy head (main site of stings), and was prescribed antihistamine (Fenergan) by intramuscular route, with subsequent progressive improvement of symptoms.
- Twelve hours after the antivenom infusion, she reported visual and auditory hallucinations. These alterations were interpreted as a “withdrawal crisis”.
- On the first day of evaluation, the patient had increased serum levels of ALT and CRP, in addition to leukocytosis and thrombocytopenia.
- ***Adverse events****:* hallucinations, numb lips and, an itchy head. The CRP levels remained elevated until the last return visit, that is, 30 days afterward.

**Participant 00111**

- **The time elapsed between the accident and infusion of apilic antivenom was four days**
- Prior to antivenom infusion, the patient presented elevated levels of CPK and CRP. At return visits, all the laboratory exams were normalized.
- ***Adverse events****:* nothing was found.

**Participant 00112**

- **The time elapsed between the accident and infusion of apilic antivenom was four days**
- ***Adverse events****:* increase of CPK levels on the day of antivenom infusion: this exam was normalized at return visits.

**Participant 00113**

- **The time elapsed between the accident and infusion of apilic antivenom was four days**
- Patient-reported arterial hypertension, chronic venous insufficiency, asthma, recurrent urinary infection, anemia, and rheumatism
- The patient returned after hospital discharge but before the first return scheduled for 10 days after complaining of: dizziness, reddish skin lesions with local burning, and itching.
- Antihistamines were prescribed for oral administration. She maintained dizziness and nausea.
- At the second return visit, that is, at 20 days afterward, the patient maintained the clinical picture of shortness of breath upon exertion, tiredness, and wheezing in the chest, but reported already having had these symptoms before the envenomation.
- ***Adverse events:*** acute urticaria, itching at the bite sites, tachycardia, and lower limb edema.

**00114 – Participant classified as a moderate case**

- **The time elapsed between the accident and infusion of apilic antivenom was one day**
- CPK, fibrinogen, CRP, and eosinophils were elevated at hospital discharge.
- At the first return visit, that is, 10 days after hospital discharge, the patient reported intense itching at the site of the stings.
- ***Adverse events:*** intense itching at sting sites. Thirty days afterward, all exam results were within normality.

**Participant 00115**

- **The time elapsed between the accident and infusion of apilic antivenom was one day**
- Presented, immediately after the antivenom infusion, coughing with tracheal secretion, in addition to tiredness and fatigue.
- There was a worsening of dermatological lesions two days after antivenom application.
- ***Adverse events:*** cough with tracheal discharge, neck pain, tiredness, and fatigue, worsening of dermatological lesions, strong odor in the urine, renal pain, and testicular pain. He presented altered CPK and PCR at hospital admission. On the return visits, these alterations disappeared.

**Participant 00116**

- **The time elapsed between the accident and infusion of apilic antivenom was one day**
- Patient reports having kidney stones and thalassemia.
- Two days after hospital discharge, the patient presented generalized intense itching and worsening of the hyperemia at the sting sites.
- At the first return visit, i.e., 10 days after antivenom application, tiredness and drowsiness were reported.
- ***Adverse events:*** itching, worsening of hyperemia, drowsiness, and tiredness.

**00117 – Participant classified as a severe case**

- **The time elapsed between the accident and infusion of apilic antivenom was six days.**
- Patient referenced generalized muscle pain and itchiness at sting sites.
- Thirty minutes after the start of the antivenom infusion, the patient presented itching of the entire trunk and the appearance of papules of approximately one centimeter compatible with an urticarial reaction of moderate intensity.
- The patient was treated with Fenergan and symptoms disappeared quickly. Next, the rest of the antivenom was applied slowly.
- Thirty days after hospital discharge, that is, at the last return visit, muscle weakness was reported.
- ***Adverse events:*** itching at sting sites, urticarial reaction.

**Participant 00301**

- **The time elapsed between the accident and infusion of apilic antivenom was less than 24 hours**
- Between the first and the second return, that is, more than 10 days after the treatment, the patient presented a clinical picture of non-specific flu.
- Was treated with Amoxicillin + Clavulanate, Prednisone, and Levocetirizine.
- ***Adverse events****:* maintenance of elevated levels of C-reactive protein at 30 days after the specific treatment.

**Participant 00302**

- **The time elapsed between the accident and infusion of apilic antivenom was less than 24 hours**
- The patient arrived at the hospital in poor general condition and with a blood pressure of 70x30 mmHg. The attending physician interpreted it as a possible onset of anaphylactic shock caused by the envenomation. In addition, the electrocardiographic examination showed cardiac arrhythmia and the lung X-ray examination revealed obliteration of the left costophrenic sinus and bilateral diffuse reticular opacity. Before the infusion of the antivenom, the medical doctor hemodynamically stabilized the patient by applying an ampoule of subcutaneous adrenaline, antihistamines, and corticosteroids. Only after the improvement of vital signs was specific treatment administered.
- ***Adverse events****:* Maintenance of elevated levels of C-reactive protein, fibrinogen, and leukocytes 30 days after the specific treatment.

**Participant 00303**

- **The time elapsed between the accident and infusion of apilic antivenom was one day.**
- Patient presented mild abdominal pain at the moment of hospital admission.
- Simethicone was prescribed.

***Adverse events*:** mild abdominal pain, maintenance of elevated CPK levels at 30 days after the specific treatment.

**Participant 00304**

- **The time elapsed between the accident and infusion of apilic antivenom was less than 24 hours**
- Patient reported being a carrier of arterial hypertension, diabetes type II, hypothyroidism, and dyslipidemia.

***Adverse events*:** elevation of blood glucose during hospitalization and maintenance of elevated fibrinogen levels 30 days after the specific treatment.
