# Supplementary material for: Single-Arm, Multicenter Phase I/II Clinical Trial for the Treatment of Envenomings by Massive Africanized Honey Bee Stings Using the Unique Apilic Antivenom
Source: Front Immunol. 2021 Mar 23;12:653151. doi: 10.3389/fimmu.2021.653151 (PMC8025786; doi:10.3389/fimmu.2021.653151)
Supplement: Supplementary file 5 [file DataSheet_5.docx]

**Supplementary Data Sheet 5 – Supplemental material of Laboratory exams results**

- **C-reactive protein (CRP)**

| **Participant** | **Estimated number of stings** | **Time between accident and clinical care** | **C-reactive protein**  **(CRP)** | | | | |  |
| --- | --- | --- | --- | --- | --- | --- | --- | --- |
|  |  |  | **B** | **D** | **R1** | **R2** | **R3** | |
| 00101 | 400 | 3 | 0,6 |  | 0,5 | 0,5 | 0,5 | |
| 00102 | 40 | 10 | 0,5 | 1,2 | 0,5 | 0,5 | 0,5 | |
| 00103 | 10 | 0 | 0,9 | 0,5 | 0,9 | 2,6 | 1,1 | |
| 00105 | 16 | 0 | 0,5 | 0,8 | 0,5 | 0,5 | 0,5 | |
| 00106 | 10 | 2 | 2 | 2,1 | 0,5 | 0,5 | 1,1 | |
| 00107 | 150 | 1 | 0,5 | 7,6 | 1 | 0,9 | 0,9 | |
| 00108 | 500 | 19 | 5,3 | 1,1 |  |  |  | |
| 00109 | 55 | 2 | 1,4 |  | 0,5 | 0,5 | 0,5 | |
| 00110 | 165 | 2 | 0,6 | 1,6 | 0,8 | 0,5 |  | |
| 00111 | 10 | 4 | 1,4 | 0,5 | 0,5 | 0,6 | 0,5 | |
| 00112 | 30 | 4 | 0,5 | 0,5 | 0,5 | 0,5 |  | |
| 00113 | 50 | 4 | 3 |  | 0,6 | 0,8 | 0,8 | |
| 00114 | 500 | 1 | 3,6 | 0,5 | 0,6 | 0,5 | 0,5 | |
| 00115 | 100 | 1 | 1,1 |  | 0,5 | 0,5 | 0,5 | |
| 00116 | 180 | 1 | 3,5 | 0,5 | 0,5 | 0,5 | 0,5 | |
| 00117 | 2000 | 6 | 0,5 | 5,4 |  |  | 0,5 | |
| 00301 | 20 | 0 | 3,6 | 3,4 | 3,4 | 1,4 | 5,4 | |
| 00302 | 150 | 0 | 3,1 | 4,7 | 3,3 | 4,8 | 9,4 | |
| 00303 | 7 | 1 | 6,5 | 2,5 | 7,1 | 5,8 | 1,6 | |
| 00304 | 50 | 0 | 2,5 |  | 1,5 | 3,2 | 3,3 | |

**B=before antivenom administration; D=discharge day; R1, R2 and R3 = 10, 20 and 30 days after discharge, respectively.**

- **Erythrocyte sedimentation rate (ESR)**

| **Participant** | **Estimated number of stings** | **Time between accident and clinical care** | **Erythrocyte sedimentation rate**  **(ESR)** | | | | |
| --- | --- | --- | --- | --- | --- | --- | --- |
|  |  |  | **B** | **D** | **R1** | **R2** | **R3** |
| 00101 | 400 | 3 | 4 |  | 5 | 16 | 8 |
| 00102 | 40 | 10 | 5 |  | 2 | 7 | 2 |
| 00103 | 10 | 0 | 19 | 10 | 28 | 17 | 46 |
| 00105 | 16 | 0 | 2 | 15 | 20 | 17 | 10 |
| 00106 | 10 | 2 | 32 | 32 | 15 | 8 | 9 |
| 00107 | 150 | 1 | 13 | 43 |  | 77 | 55 |
| 00108 | 500 | 19 | 16 |  |  |  |  |
| 00109 | 55 | 2 |  | 16 | 10 | 10 | 7 |
| 00110 | 165 | 2 |  |  | 18 | 13 | 9 |
| 00111 | 10 | 4 | 38 | 52 | 31 | 21 | 17 |
| 00112 | 30 | 4 | 4 | 9 | 14 | 14 |  |
| 00113 | 50 | 4 | 12 | 4 | 3 | 8 | 10 |
| 00114 | 500 | 1 | 19 | 24 | 12 | 16 | 10 |
| 00115 | 100 | 1 | 2 | 2 | 2 | 2 | 5 |
| 00116 | 180 | 1 | 5 |  | 3 | 6 | 6 |
| 00117 | 2000 | 6 |  | 2 |  |  | 6 |
| 00301 | 20 | 0 | 10 | 10 | 12 | 20 | 30 |
| 00302 | 150 | 0 | 25 | 14 | 61 | 40 | 80 |
| 00303 | 7 | 1 | 16 | 30 | 5 | 30 | 4 |
| 00304 | 50 | 0 | 50 |  | 88 |  |  |

**B=before antivenom administration; D=discharge day; R1, R2 and R3 = 10, 20 and 30 days after discharge, respectively.**

- **Creatine kinase (CK)**

| **Participant** | **Estimated number of stings** | **Time between accident and clinical care** | **Creatine phosphokinase**  **(CPK)** | | | | |
| --- | --- | --- | --- | --- | --- | --- | --- |
|  |  |  | **B** | **D** | **R1** | **R2** | **R3** |
| 00101 | 400 | 3 | 364 |  | 55 | 74 | 59 |
| 00102 | 40 | 10 | 88 |  | 158 | 106 | 160 |
| 00103 | 10 | 0 | 708 | 520 | 253 | 194 | 516 |
| 00105 | 16 | 0 | 172 | 118 | 165 | 131 | 113 |
| 00106 | 10 | 2 | 40 | 23 | 1 | 33 | 42 |
| 00107 | 150 | 1 | 189 | 74 | 196 | 62 | 90 |
| 00108 | 500 | 19 | 53 | 163 |  |  |  |
| 00109 | 55 | 2 | 823 | 1153 | 227 | 119 | 87 |
| 00110 | 165 | 2 | 545 |  | 81 | 88 | 109 |
| 00111 | 10 | 4 | 52 | 28 | 43 | 60 | 59 |
| 00112 | 30 | 4 | 354 | 151 | 157 | 248 |  |
| 00113 | 50 | 4 | 1136 | 273 | 60 | 67 | 78 |
| 00114 | 500 | 1 | 11504 | 7541 | 59 | 68 | 89 |
| 00115 | 100 | 1 | 271 | 240 | 173 | 138 | 106 |
| 00116 | 180 | 1 | 424 |  | 65 | 54 | 76 |
| 00117 | 2000 | 6 | 1222 | 544 |  |  | 161 |
| 00301 | 20 | 0 | 188 | 109 | 378 | 104 | 78 |
| 00302 | 150 | 0 | 66 | 38 | 65 | 161 | 50 |
| 00303 | 7 | 1 | 196 | 114 | 290 | 170 | 219 |
| 00304 | 50 | 0 | 66 | 66 | 19 | 39 | 44 |

**B=before antivenom administration; D=discharge day; R1, R2 and R3 = 10, 20 and 30 days after discharge, respectively.**

- **Creatinine (Cr)**

| **Participant** | **Estimated number of stings** | **Time between accident and clinical care** | **Creatinine**  **(Cr)** | | | | |
| --- | --- | --- | --- | --- | --- | --- | --- |
|  |  |  | **B** | **D** | **R1** | **R2** | **R3** |
| 00101 | 400 | 3 | 0.8 | 0.7 |  | 0.7 | 0.6 |
| 00101 | 400 | 3 | 0.8 | 0.7 |  | 0.7 | 0.6 |
| 00102 | 40 | 10 | 1 | 0.9 |  | 0.9 | 0.9 |
| 00103 | 10 | 0 | 1.3 |  | 0.9 | 1 | 1 |
| 00105 | 16 | 0 | 1 |  | 1 | 1 | 1 |
| 00106 | 10 | 2 | 1 |  | 0.7 | 0.6 | 0.8 |
| 00107 | 150 | 1 | 0.9 |  | 0.8 | 0.7 | 0.7 |
| 00108 | 500 | 19 | 1 |  | 1 |  |  |
| 00109 | 55 | 2 | 2.4 |  | 1.1 | 0.8 | 0.8 |
| 00110 | 165 | 2 | 0.7 | 0.6 |  | 0.7 | 0.6 |
| 00111 | 10 | 4 | 0.6 |  | 0.6 | 0.7 | 0.6 |
| 00112 | 30 | 4 | 0.7 |  | 0.7 | 0.7 | 0.7 |
| 00113 | 50 | 4 | 0.9 | 0.7 | 0.8 | 0.8 | 0.8 |
| 00114 | 500 | 1 | 1 | 0.9 | 1 | 1 | 1 |
| 00115 | 100 | 1 | 1.1 |  | 1.1 | 0.9 | 1 |
| 00116 | 180 | 1 | 0.8 |  |  | 0.9 | 0.8 |
| 00117 | 2000 | 6 | 1 |  | 1.1 |  |  |
| 00301 | 20 | 0 | 1.25 |  | 1.18 | 1.14 | 0.98 |
| 00302 | 150 | 0 | 0.82 | 1.1 | 0.9 | 1.08 | 1.35 |
| 00303 | 7 | 1 | 0.83 |  | 0.87 | 0.92 | 0.76 |
| 00304 | 50 | 0 | 0.78 |  |  | 0.69 | 0.72 |

**B=before antivenom administration; D=discharge day; R1, R2 and R3 = 10, 20 and 30 days after discharge, respectively.**

- **Fibrinogen (Fg)**

| **Participant** | **Estimated number of stings** | **Time between accident and clinical care** | **Fibrinogen**  **(Fg)** | | | | |  |
| --- | --- | --- | --- | --- | --- | --- | --- | --- |
|  |  |  | **B** | **D** | **R1** | **R2** | **R3** | |
| 00101 | 400 | 3 | 259 | 189 |  | 279 | 306 | |
| 00101 | 400 | 3 | 259 | 189 |  | 279 | 306 | |
| 00102 | 40 | 10 | 199 | 210 |  | 190 | 261 | |
| 00103 | 10 | 0 | 358 |  | 342 | 362 | 380 | |
| 00105 | 16 | 0 | 310 |  | 323 | 326 |  | |
| 00106 | 10 | 2 | 376 |  | 299 | 214 | 287 | |
| 00107 | 150 | 1 | 414 |  | 492 | 498 | 510 | |
| 00108 | 500 | 19 | 543 |  | 670 |  |  | |
| 00109 | 55 | 2 | 360 |  | 506 | 359 | 330 | |
| 00110 | 165 | 2 |  | 434 |  | 402 | 363 | |
| 00111 | 10 | 4 | 231 |  | 313 | 232 | 250 | |
| 00112 | 30 | 4 | 286 |  | 291 | 348 | 321 | |
| 00113 | 50 | 4 | 373 | 276 | 326 | 272 | 307 | |
| 00114 | 500 | 1 | 401 | 379 | 401 |  | 317 | |
| 00115 | 100 | 1 | 243 |  | 262 | 180 | 178 | |
| 00116 | 180 | 1 | 394 |  |  | 230 |  | |
| 00117 | 2000 | 6 |  |  |  |  |  | |
| 00301 | 20 | 0 | 306 |  | 285 | 273 | 266 | |
| 00302 | 150 | 0 | 407 | 392 | 335 | 341 | 285 | |
| 00303 | 7 | 1 | 335 |  | 360 | 335 | 360 | |
| 00304 | 50 | 0 | 419 |  | 401 | 419 | 393 | |

**B=before antivenom administration; D=discharge day; R1, R2 and R3 = 10, 20 and 30 days after discharge, respectively.**

- **Alanine aminotransferase (ALT)**

| **Participant** | **Estimated number of stings** | **Time between accident and clinical care** | **Alanine aminotransferase**  **(ALT)** | | | | |
| --- | --- | --- | --- | --- | --- | --- | --- |
|  |  |  | **B** | **D** | **R1** | **R2** | **R3** |
| 00101 | 400 | 3 | 140 | 101 |  | 52 | 35 |
| 00101 | 400 | 3 | 140 | 101 |  | 52 | 35 |
| 00102 | 40 | 10 | 34 | 28 |  | 32 | 24 |
| 00103 | 10 | 0 | 40 |  | 35 | 31 | 35 |
| 00105 | 16 | 0 | 56 |  | 58 | 73 | 66 |
| 00106 | 10 | 2 | 54 |  | 34 | 23 | 21 |
| 00107 | 150 | 1 | 31 |  | 24 | 25 | 27 |
| 00108 | 500 | 19 | 97 |  | 84 |  |  |
| 00109 | 55 | 2 | 43 |  | 59 | 26 | 27 |
| 00110 | 165 | 2 | 37 | 42 |  | 38 | 37 |
| 00111 | 10 | 4 | 20 |  | 26 | 17 | 19 |
| 00112 | 30 | 4 | 33 |  |  |  |  |
| 00113 | 50 | 4 | 48 | 128 | 111 | 50 | 39 |
| 00114 | 500 | 1 | 60 | 57 | 69 | 75 | 27 |
| 00115 | 100 | 1 | 43 |  | 50 | 28 | 30 |
| 00116 | 180 | 1 | 38 |  |  | 53 | 28 |
| 00117 | 2000 | 6 | 404 |  | 277 |  |  |
| 00301 | 20 | 0 | 11 |  | 12 | 16 | 20 |
| 00302 | 150 | 0 | 12 | 14 | 12 | 10 | 12 |
| 00303 | 7 | 1 | 15 |  | 13 | 20 | 18 |
| 00304 | 50 | 0 | 14 |  |  | 14 | 15 |

**B=before antivenom administration; D=discharge day; R1, R2 and R3 = 10, 20 and 30 days after discharge, respectively.**

- **Leukocytes (Leu)**

| **Participant** | **Estimated number of stings** | **Time between accident and clinical care** | **Leukocytes**  **(Leu) (*)** | | | | | | | | |
| --- | --- | --- | --- | --- | --- | --- | --- | --- | --- | --- | --- |
|  |  |  | **B** | | **D** | | **R1** | | **R2** | **R3** | |
| 00101 | 400 | 3 | 14.7 | 10.0 | |  | | 10.7 | | | 5.3 |
| 00101 | 400 | 3 | 14.7 | 10.0 | |  | | 10.7 | | | 5.3 |
| 00102 | 40 | 10 | 9.4 | 7.8 | |  | | 6 | | | 8.1 |
| 00103 | 10 | 0 | 7.2 |  | | 0.7 | | 5.7 | | | 7 |
| 00105 | 16 | 0 | 5.3 |  | | 6.5 | | 6 | | | 5.9 |
| 00106 | 10 | 2 | 11.2 |  | |  | | 6.5 | | | 8.2 |
| 00107 | 150 | 1 | 19.5 |  | | 8.4 | | 9.6 | | | 9 |
| 00108 | 500 | 19 | 7.8 |  | | 10.3 | |  | | |  |
| 00109 | 55 | 2 | 17.4 |  | | 7.9 | | 7.3 | | | 24.5 |
| 00110 | 165 | 2 | 15.2 | 10.3 | |  | | 7.9 | | | 8.9 |
| 00111 | 10 | 4 | 10.2 |  | | 9 | | 6.3 | | | 5.9 |
| 00112 | 30 | 4 | 5.6 |  | | 6.4 | | 6.1 | | | 7.9 |
| 00113 | 50 | 4 | 13.3 | 8.1 | | 6.9 | | 7.1 | | | 8.4 |
| 00114 | 500 | 1 | 17.1 | 9.3 | | 7.7 | | 9.6 | | | 3.9 |
| 00115 | 100 | 1 | 9.4 |  | | 6.4 | | 4.7 | | | 7.6 |
| 00116 | 180 | 1 | 10.5 |  | |  | | 8.2 | | | 5.8 |
| 00117 | 2000 | 6 | 11.2 |  | |  | |  | | |  |
| 00301 | 20 | 0 | 10.1 |  | | 6.9 | | 6.8 | | | 5.6 |
| 00302 | 150 | 0 | 17.6 | 11.6 | | 17.53 | | 11.7 | | | 7.61 |
| 00303 | 7 | 1 | 9.3 |  | | 8.9 | | 13.62 | | | 10.2 |
| 00304 | 50 | 0 | 10.3 |  | |  | | 4 | | | 3.1 |

**B=before antivenom administration; D=discharge day; R1, R2 and R3 = 10, 20 and 30 days after discharge, respectively.**

**(*) All numbers of Leukocytes are multiplied by 10^3^.**

- **Platelets (Plt)**

| **Participant** | **Estimated number of stings** | **Time between accident and clinical care** | **Platelets**  **(Plt) (*)** | | | | | | | |  |  |
| --- | --- | --- | --- | --- | --- | --- | --- | --- | --- | --- | --- | --- |
|  |  |  | **B** | **D** | | **R1** | | **R2** | **R3** | | |  |
| 00101 | 400 | 3 | 347 | 321 |  | | 379 | | | 335 | | |
| 00101 | 400 | 3 | 347 | 321 |  | | 379 | | | 335 | | |
| 00102 | 40 | 10 | 196 | 187 |  | | 157 | | | 171 | | |
| 00103 | 10 | 0 | 277 |  | 201 | | 218 | | | 239 | | |
| 00105 | 16 | 0 | 252 |  | 284 | | 319 | | | 278 | | |
| 00106 | 10 | 2 | 638 |  |  | | 476 | | | 349 | | |
| 00107 | 150 | 1 | 410 |  | 408 | | 408 | | | 387 | | |
| 00108 | 500 | 19 | 186 |  | 197 | |  | | |  | | |
| 00109 | 55 | 2 | 158 |  | 123 | | 217 | | | 180 | | |
| 00110 | 165 | 2 | 211 | 203 |  | | 438 | | | 288 | | |
| 00111 | 10 | 4 | 312 |  | 342 | | 270 | | | 300 | | |
| 00112 | 30 | 4 | 267 |  | 299 | | 259 | | | 312 | | |
| 00113 | 50 | 4 | 320 | 352 | 310 | | 342 | | | 377 | | |
| 00114 | 500 | 1 | 213 | 181 | 206 | | 277 | | | 233 | | |
| 00115 | 100 | 1 | 213 |  | 176 | | 224 | | | 252 | | |
| 00116 | 180 | 1 | 269 |  |  | | 331 | | | 307 | | |
| 00117 | 2000 | 6 | 190 |  |  | |  | | |  | | |
| 00301 | 20 | 0 | 297 |  | 298 | | 291 | | | 234 | | |
| 00302 | 150 | 0 | 271 | 293 | 297 | | 336 | | | 339 | | |
| 00303 | 7 | 1 | 240 |  | 219 | | 201 | | | 211 | | |
| 00304 | 50 | 0 | 302 |  |  | | 209 | | | 283 | | |

**B=before antivenom administration; D=discharge day; R1, R2 and R3 = 10, 20 and 30 days after discharge, respectively.**

**(*) All numbers of Platelets are multiplied by 10^3^.**

- **Hematocrit (Ht)**

| **Participant** | **Estimated number of stings** | **Time between accident and clinical care** | **Hematocrit**  **(Ht)** | | | | | | | | |
| --- | --- | --- | --- | --- | --- | --- | --- | --- | --- | --- | --- |
|  |  |  | **B** | | **D** | | **R1** | | **R2** | **R3** | |
| 00101 | 400 | 3 | 37.8 | 34.3 | |  | | 41.9 | | | 40.7 |
| 00101 | 400 | 3 | 37.8 | 34.3 | |  | | 41.9 | | | 40.7 |
| 00102 | 40 | 10 | 45.5 | 45 | |  | | 43 | | | 44.1 |
| 00103 | 10 | 0 | 43.1 |  | | 40.3 | | 43.5 | | | 43 |
| 00105 | 16 | 0 | 47.8 |  | | 50.6 | | 48.7 | | | 45.6 |
| 00106 | 10 | 2 | 36.2 |  | |  | | 34.6 | | | 35.3 |
| 00107 | 150 | 1 | 46.4 |  | | 39.6 | | 38 | | | 38.9 |
| 00108 | 500 | 19 | 43.1 |  | | 55.5 | |  | | |  |
| 00109 | 55 | 2 | 50.4 |  | | 44.5 | | 44.4 | | | 46.3 |
| 00110 | 165 | 2 | 41.8 | 35.3 | |  | | 36.5 | | | 38.6 |
| 00111 | 10 | 4 | 38.3 |  | | 36.7 | | 36.8 | | | 36.3 |
| 00112 | 30 | 4 | 35.3 |  | | 36.7 | | 35.3 | | | 36.2 |
| 00113 | 50 | 4 | 36.3 | 37.1 | | 35 | | 36.6 | | | 39.5 |
| 00114 | 500 | 1 | 39.4 | 35.5 | | 38 | | 43.3 | | | 41.2 |
| 00115 | 100 | 1 | 45 |  | | 46.6 | | 46 | | | 46.4 |
| 00116 | 180 | 1 | 38.97 |  | |  | | 40.8 | | | 37.4 |
| 00117 | 2000 | 6 | 44.7 |  | |  | |  | | |  |
| 00301 | 20 | 0 | 45.4 |  | | 45.6 | | 47 | | | 44.8 |
| 00302 | 150 | 0 | 36 | 37.4 | | 38.7 | | 33.8 | | | 36.7 |
| 00303 | 7 | 1 | 42.7 |  | | 38.8 | | 41.5 | | | 44.1 |
| 00304 | 50 | 0 | 39.3 |  | |  | | 34.8 | | | 36.2 |

**B=before antivenom administration; D=discharge day; R1, R2 and R3 = 10, 20 and 30 days after discharge, respectively.**

- **Haemoglobin (Hb)**

| **Participant** | **Estimated number of stings** | **Time between accident and clinical care** | **Haemoglobin**  **(Hb)** | | | | | | | |
| --- | --- | --- | --- | --- | --- | --- | --- | --- | --- | --- |
|  |  |  | **B** | | **D** | | **R1** | | **R2** | **R3** |
| 00101 | 400 | 3 | 12.8 | 12 | |  | | 14.7 | | 13 |
| 00101 | 400 | 3 | 12.8 | 12 | |  | | 14.7 | | 13 |
| 00102 | 40 | 10 | 15.4 | 15.3 | |  | | 15 | | 14.9 |
| 00103 | 10 | 0 | 14.4 |  | | 13.1 | | 14.2 | | 14.6 |
| 00105 | 16 | 0 | 15.9 |  | | 15.9 | | 16.2 | | 15.7 |
| 00106 | 10 | 2 | 12 |  | |  | | 11.7 | | 12 |
| 00107 | 150 | 1 | 15.7 |  | | 13.4 | | 13 | | 12.9 |
| 00108 | 500 | 19 | 14.8 |  | | 18.3 | |  | |  |
| 00109 | 55 | 2 | 17.6 |  | | 15.8 | | 14.9 | | 16.1 |
| 00110 | 165 | 2 | 14.69 | 12.3 | |  | | 12.2 | | 13.2 |
| 00111 | 10 | 4 | 12.9 |  | | 12.2 | | 11.8 | | 12.7 |
| 00112 | 30 | 4 | 11.9 |  | | 12.5 | | 11.5 | | 12.4 |
| 00113 | 50 | 4 | 11.8 | 11.8 | | 11.3 | | 11.4 | | 13.2 |
| 00114 | 500 | 1 | 13.6 | 12.3 | | 13.8 | | 15.7 | | 13.6 |
| 00115 | 100 | 1 | 15.6 |  | | 15.9 | | 15.8 | | 15.3 |
| 00116 | 180 | 1 | 12.5 |  | |  | | 12.3 | | 11.4 |
| 00117 | 2000 | 6 | 15 |  | |  | |  | |  |
| 00301 | 20 | 0 | 14.9 |  | | 14.9 | | 15.3 | | 14.6 |
| 00302 | 150 | 0 | 11.6 | 12.1 | | 12.5 | | 10.8 | | 11.5 |
| 00303 | 7 | 1 | 14.7 |  | | 13.7 | | 14.1 | | 15.2 |
| 00304 | 50 | 0 | 13.5 |  | |  | | 12.4 | | 12.3 |

**B=before antivenom administration; D=discharge day; R1, R2 and R3 = 10, 20 and 30 days after discharge, respectively.**
