# Supplementary material for: Single-Arm, Multicenter Phase I/II Clinical Trial for the Treatment of Envenomings by Massive Africanized Honey Bee Stings Using the Unique Apilic Antivenom
Source: Front Immunol. 2021 Mar 23;12:653151. doi: 10.3389/fimmu.2021.653151 (PMC8025786; doi:10.3389/fimmu.2021.653151)
Supplement: Supplementary file 6 [file DataSheet_6.docx]

**Supplementary Data Sheet 6 – Enzyme-Linked Immunosorbent Assay**

Table 1 shows the absorbance average under different concentrations of bee venom to calculate the straight-line equation (Figure 1). Figures 2 to 19 show *Apis mellifera* venom (melittin and phospholipase A_2_) quantification in the blood of each participant.

**Table 1:** Absorbance average (nm) of the calibrators under different concentrations of the Africanized bee venom (*Apis mellifera*).

| **Absorbance average (nm)** | **Concentration (ng/mL)** |
| --- | --- |
|  | |
| 1,1225 | 10000 |
| 1,1075 | 5000 |
| 1,0135 | 2000 |
| 0,995 | 1000 |
| 0,5445 | 500 |
| 0,236 | 200 |
| 0,152 | 100 |
| 0,0885 | 50 |
| 0,072 | 20 |
| 0,0915 | 10 |
| 0,1105 | 5 |
| 0,154 | 2 |
| 0,158 | 1 |

**
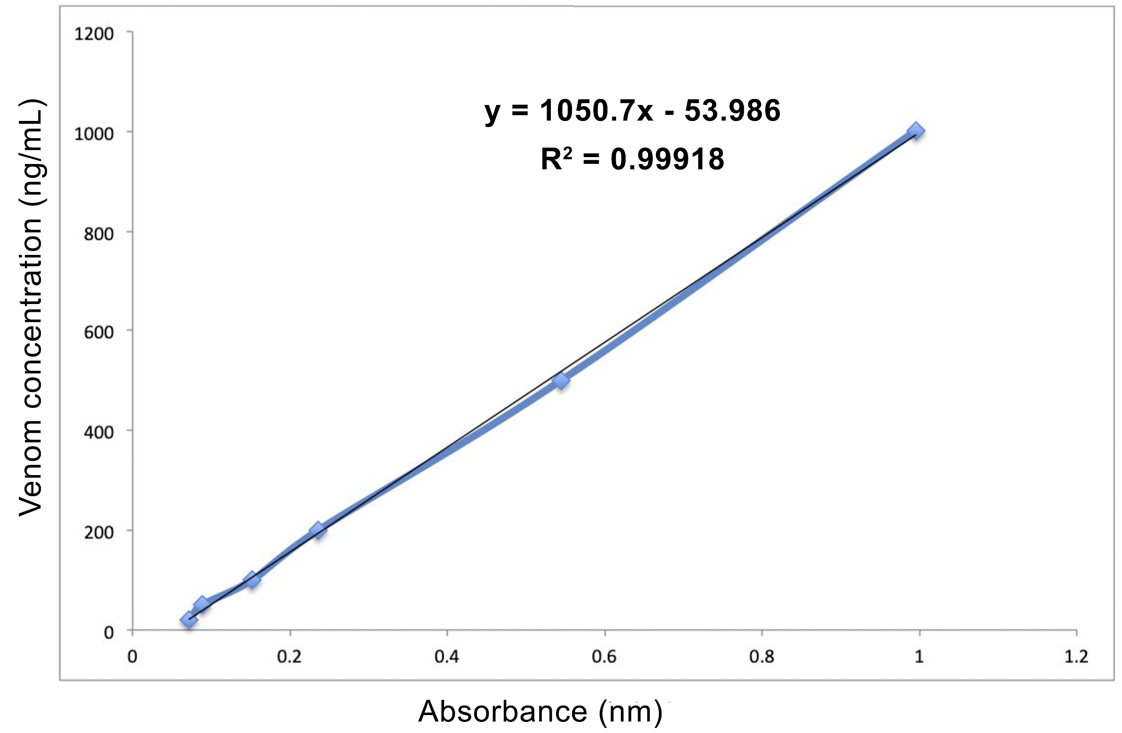
**

**Figure 1:** Calibrators curve using concentrations from 20 to 1000 ng/mL of Africanized bee venom *- Apis mellifera*. (y= 1050,7x-53.986, R2= 0,99918).


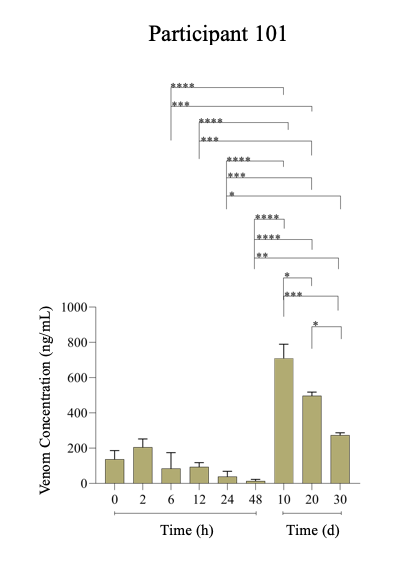


**Figure 2.** *Apis mellifera* venom quantification in the blood of participant 101 using the Enzyme-Linked Immunosorbent Assay (ELISA). Time 0 corresponds to the participant’s admission to the hospital service. Times 2, 6, 12, 24 and 48 hours refer to the time after the therapeutic intervention using the apilic antivenom. Times 10, 20 and 30 days correspond to the participant's return to hospital service after discharge. The values represent the mean ± standard deviation of the absorbances of each biological replica analyzed. The results obtained were compared using the ANOVA test for repeated measures followed by the Tukey test (* p <0.1; ** p <0.01; *** p <0.001 and **** p <0.0001).


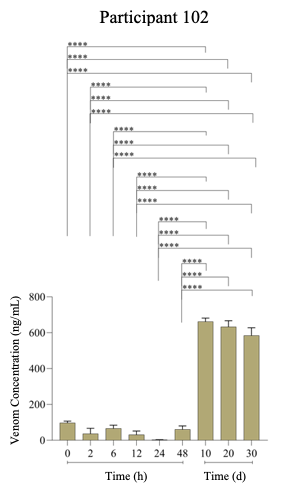


**Figure 3.** *Apis mellifera* venom quantification in the blood of participant 102 using the Enzyme-Linked Immunosorbent Assay (ELISA). Time 0 corresponds to the participant’s admission to the hospital service. Times 2, 6, 12, 24 and 48 hours refer to the time after the therapeutic intervention using the apilic antivenom. Times 10, 20 and 30 days correspond to the participant's return to hospital service after discharge. The values represent the mean ± standard deviation of the absorbances of each biological replica analyzed. The results obtained were compared using the ANOVA test for repeated measures followed by the Tukey test (**** p <0.0001).


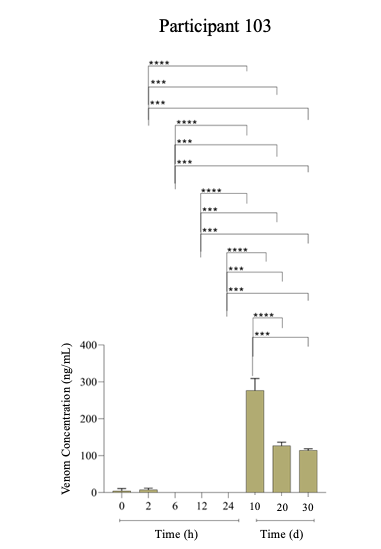


**Figure 4.** *Apis mellifera* venom quantification in the blood of participant 103 using the Enzyme-Linked Immunosorbent Assay (ELISA). Time 0 corresponds to the participant’s admission to the hospital service. Times 2, 6, 12 and 24 hours refer to the time after the therapeutic intervention using the apilic antivenom. Times 10, 20 and 30 days correspond to the participant's return to hospital service after discharge. The values represent the mean ± standard deviation of the absorbances of each biological replica analyzed. The results obtained were compared using the ANOVA test for repeated measures followed by the Tukey test (*** p <0.001 and **** p <0.0001).

**
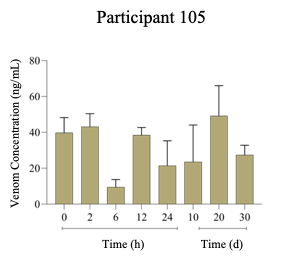
**

**Figure 5.** *Apis mellifera* venom quantification in the blood of participant 105 using the Enzyme-Linked Immunosorbent Assay (ELISA). Time 0 corresponds to the participant’s admission to the hospital service. Times 2, 6, 12 and 24 hours refer to the time after the therapeutic intervention using the apilic antivenom. Times 10, 20 and 30 days correspond to the participant's return to hospital service after discharge. The values represent the mean ± standard deviation of the absorbances of each biological replica analyzed. The results obtained were compared using the ANOVA test for repeated measures followed by the Tukey test. There was no statistical difference between the samples analyzed.

**
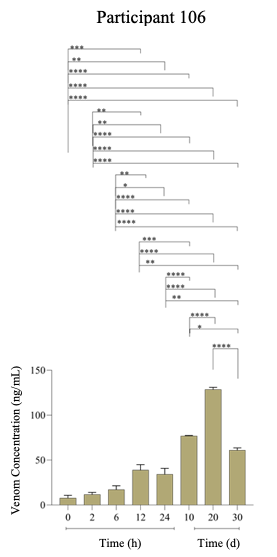
**

**Figure 6.** *Apis mellifera* venom quantification in the blood of participant 106 using the Enzyme-Linked Immunosorbent Assay (ELISA). Time 0 corresponds to the participant’s admission to the hospital service. Times 2, 6, 12 and 24 hours refer to the time after the therapeutic intervention using the apilic antivenom. Times 10, 20 and 30 days correspond to the participant's return to hospital service after discharge. The values represent the mean ± standard deviation of the absorbances of each biological replica analyzed. The results obtained were compared using the ANOVA test for repeated measures followed by the Tukey test (* p <0.1; ** p <0.01; *** p <0.001 and **** p <0.0001).

**
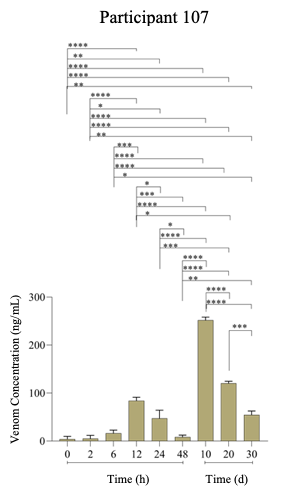
**

**Figure 7.** *Apis mellifera* venom quantification in the blood of participant 107 using the Enzyme-Linked Immunosorbent Assay (ELISA). Time 0 corresponds to the participant’s admission to the hospital service. Times 2, 6, 12, 24 and 48 hours refer to the time after the therapeutic intervention using the apilic antivenom. Times 10, 20 and 30 days correspond to the participant's return to hospital service after discharge. The values represent the mean ± standard deviation of the absorbances of each biological replica analyzed. The results obtained were compared using the ANOVA test for repeated measures followed by the Tukey test (* p <0.1; ** p <0.01; *** p <0.001 and **** p <0.0001).

**
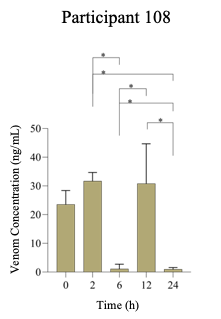
**

**Figure 8.** *Apis mellifera* venom quantification in the blood of participant 108 using the Enzyme-Linked Immunosorbent Assay (ELISA). Time 0 corresponds to the participant’s admission to the hospital service. Times 2, 6, 12 and 24 hours refer to the time after the therapeutic intervention using the apilic antivenom. The values represent the mean ± standard deviation of the absorbances of each biological replica analyzed. The results obtained were compared using the ANOVA test for repeated measures followed by the Tukey test (* p <0.1).

**
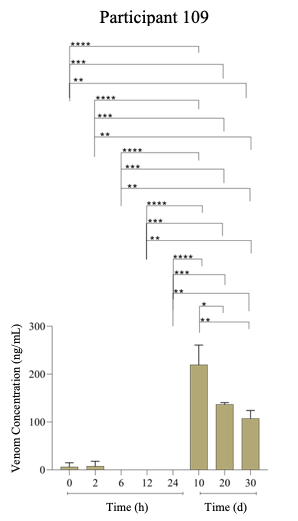
**

**Figure 9.** *Apis mellifera* venom quantification in the blood of participant 109 using the Enzyme-Linked Immunosorbent Assay (ELISA). Time 0 corresponds to the participant’s admission to the hospital service. Times 2, 6, 12 and 24 hours refer to the time after the therapeutic intervention using the apilic antivenom. Times 10, 20 and 30 days correspond to the participant's return to hospital service after discharge. The values represent the mean ± standard deviation of the absorbances of each biological replica analyzed. The results obtained were compared using the ANOVA test for repeated measures followed by the Tukey test (* p <0.1; ** p <0.01; *** p <0.001 and **** p <0.0001).

**
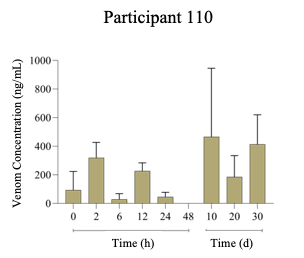
**

**Figure 10.** *Apis mellifera* venom quantification in the blood of participant 110 using the Enzyme-Linked Immunosorbent Assay (ELISA). Time 0 corresponds to the participant’s admission to the hospital service. Times 2, 6, 12, 24 and 48 hours refer to the time after the therapeutic intervention using the apilic antivenom. Times 10, 20 and 30 days correspond to the participant's return to hospital service after discharge. The values represent the mean ± standard deviation of the absorbances of each biological replica analyzed. The results obtained were compared using the ANOVA test for repeated measures followed by the Tukey test. There was no statistical difference between the samples analyzed.

**
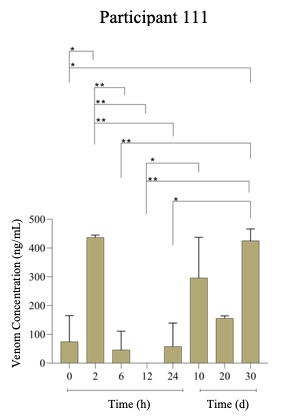
**

**Figure 11.** *Apis mellifera* venom quantification in the blood of participant 111 using the Enzyme-Linked Immunosorbent Assay (ELISA). Time 0 corresponds to the participant’s admission to the hospital service. Times 2, 6, 12 and 24 hours refer to the time after the therapeutic intervention using the apilic antivenom. Times 10, 20 and 30 days correspond to the participant's return to hospital service after discharge. The values represent the mean ± standard deviation of the absorbances of each biological replica analyzed. The results obtained were compared using the ANOVA test for repeated measures followed by the Tukey test (* p <0.1 and ** p <0.01).

**
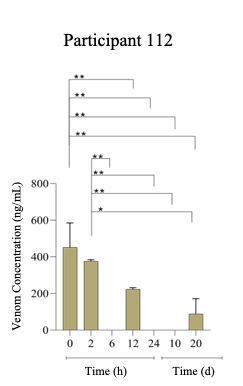
**

**Figure 12.** *Apis mellifera* venom quantification in the blood of participant 112 using the Enzyme-Linked Immunosorbent Assay (ELISA). Time 0 corresponds to the participant’s admission to the hospital service. Times 2, 6, 12 and 24 hours refer to the time after the therapeutic intervention using the apilic antivenom. Times 10 and 20 days correspond to the participant's return to hospital service after discharge. The values represent the mean ± standard deviation of the absorbances of each biological replica analyzed. The results obtained were compared using the ANOVA test for repeated measures followed by the Tukey test (* p <0.1 and ** p <0.01).

**
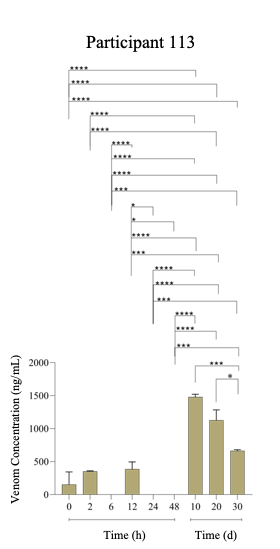
**

**Figure 13.** *Apis mellifera* venom quantification in the blood of participant 113 using the Enzyme-Linked Immunosorbent Assay (ELISA). Time 0 corresponds to the participant’s admission to the hospital service. Times 2, 6, 12, 24 and 48 hours refer to the time after the therapeutic intervention using the apilic antivenom. Times 10, 20 and 30 days correspond to the participant's return to hospital service after discharge. The values represent the mean ± standard deviation of the absorbances of each biological replica analyzed. The results obtained were compared using the ANOVA test for repeated measures followed by the Tukey test (* p <0.1; *** p <0.001 and **** p <0.0001).

**
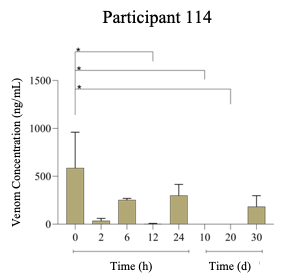
**

**Figure 14.** *Apis mellifera* venom quantification in the blood of participant 114 using the Enzyme-Linked Immunosorbent Assay (ELISA). Time 0 corresponds to the participant’s admission to the hospital service. Times 2, 6, 12 and 24 hours refer to the time after the therapeutic intervention using the apilic antivenom. Times 10, 20 and 30 days correspond to the participant's return to hospital service after discharge. The values represent the mean ± standard deviation of the absorbances of each biological replica analyzed. The results obtained were compared using the ANOVA test for repeated measures followed by the Tukey test (* p <0.1).

**
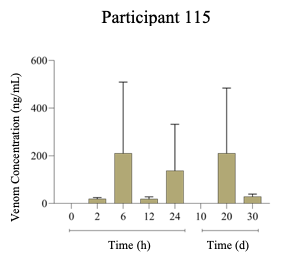
**

**Figure 15.** *Apis mellifera* venom quantification in the blood of participant 115 using the Enzyme-Linked Immunosorbent Assay (ELISA). Time 0 corresponds to the participant’s admission to the hospital service. Times 2, 6, 12 and 24 hours refer to the time after the therapeutic intervention using the apilic antivenom. Times 10, 20 and 30 days correspond to the participant's return to hospital service after discharge. The values represent the mean ± standard deviation of the absorbances of each biological replica analyzed. The results obtained were compared using the ANOVA test for repeated measures followed by the Tukey test. There was no statistical difference between the samples analyzed.

**
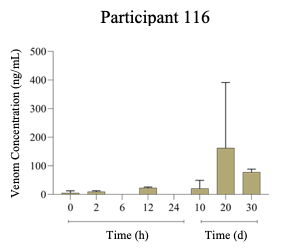
**

**Figure 16.** *Apis mellifera* venom quantification in the blood of participant 115 using the Enzyme-Linked Immunosorbent Assay (ELISA). Time 0 corresponds to the participant’s admission to the hospital service. Times 2, 6, 12 and 24 hours refer to the time after the therapeutic intervention using the apilic antivenom. Times 10, 20 and 30 days correspond to the participant's return to hospital service after discharge. The values represent the mean ± standard deviation of the absorbances of each biological replica analyzed. The results obtained were compared using the ANOVA test for repeated measures followed by the Tukey test. There was no statistical difference between the samples analyzed.

**
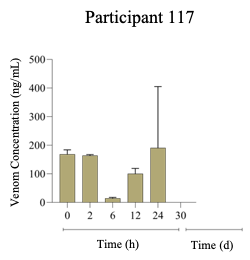
**

**Figure 17.** *Apis mellifera* venom quantification in the blood of participant 117 using the Enzyme-Linked Immunosorbent Assay (ELISA). Time 0 corresponds to the participant’s admission to the hospital service. Times 2, 6, 12 and 24 hours refer to the time after the therapeutic intervention using the apilic antivenom. Time 30 days correspond to the participant's return to hospital service after discharge. The values represent the mean ± standard deviation of the absorbances of each biological replica analyzed. The results obtained were compared using the ANOVA test for repeated measures followed by the Tukey test. There was no statistical difference between the samples analyzed.

**
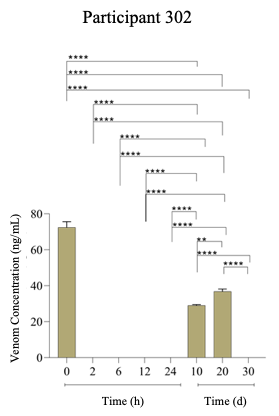
**

**Figure 18.** *Apis mellifera* venom quantification in the blood of participant 302 using the Enzyme-Linked Immunosorbent Assay (ELISA). Time 0 corresponds to the participant’s admission to the hospital service. Times 2, 6, 12 and 24 hours refer to the time after the therapeutic intervention using the apilic antivenom. Times 10, 20 and 30 days correspond to the participant's return to hospital service after discharge. The values represent the mean ± standard deviation of the absorbances of each biological replica analyzed. The results obtained were compared using the ANOVA test for repeated measures followed by the Tukey test (** p <0.01 and **** p <0.0001).

**
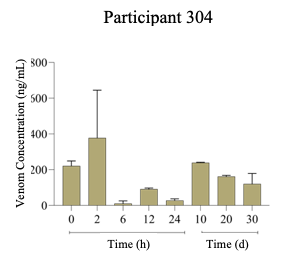
**

**Figure 19.** *Apis mellifera* venom quantification in the blood of participant 304 using the Enzyme-Linked Immunosorbent Assay (ELISA). Time 0 corresponds to the participant’s admission to the hospital service. Times 2, 6, 12 and 24 hours refer to the time after the therapeutic intervention using the apilic antivenom. Times 10, 20 and 30 days correspond to the participant's return to hospital service after discharge. The values represent the mean ± standard deviation of the absorbances of each biological replica analyzed. The results obtained were compared using the ANOVA test for repeated measures followed by the Tukey test. There was no statistical difference between the samples analyzed.

**Table 2.** Level of venom concentration (melittin and phospholipase A2) during periods evaluated

| Patient / Time | **101** | **102** | **103** | **105** | **106** | **107** | **108** | **109** | **110** | **111** | **112** | **113** | **114** | **115** | **116** | **117** | **302** | **304** |
| --- | --- | --- | --- | --- | --- | --- | --- | --- | --- | --- | --- | --- | --- | --- | --- | --- | --- | --- |
| **0 h** | 136,8411 | 97,9652 | 0,03391 | 39,78104 | 7,79054 | 0 | 23,57252 | 0,88157 | 91,9224 | 74,3826 | 452,4204 | 153,2085 | 587,3591 | 0 | 0 | 168,4445 | 72,44548 | 221,2565 |
| **2 h** | 205,1366 | 37,0246 | 7,66285 | 43,19336 | 11,6294 | 5,2313 | 31,67678 | 7,66285 | 319,9398 | 436,8718 | 376,1507 | 352,6831 | 35,8705 | 19,3433 | 9,78002 | 164,0435 | 0 | 377,492 |
| **6 h** | 84,3061 | 66,4442 | 0 | 9,4967 | 17,17442 | 16,32134 | 0,53936 | 0 | 0 | 0 | 0 | 0 | 252,9458 | 193,87316 | 0 | 14,4095 | 0 | 7,808 |
| **12 h** | 93,7624 | 30,7204 | 0 | 38,50142 | 43,19336 | 84,1412 | 30,8237 | 0 | 319,9398 | 0 | 223,6113 | 387,8845 | 0 | 18,54636 | 22,53106 | 100,229 | 0 | 91,427 |
| **24 h** | 39,126 | 0,2501 | 0 | 21,43982 | 34,23602 | 47,45876 | 0,9659 | 0 | 45,1496 | 45,1496 | 0 | 0 | 299,881 | 118,9608 | 0 | 190,4495 | 0 | 27,6125 |
| **48 h** | 13,9092 | 61,1907 | - | - | - | 8,64362 | - | - | 0 | - | - | 0 | 0 | - | - | - | - | - |
| **10 d** | 709,4726 | 662,1911 | 276,7949 | 23,57252 | 76,89002 | 251,77142 | - | 219,57785 | 466,1048 | 296,5534 | 0 | 1479,1279 | 0 | 0 | 16,15554 | - | 29,03484 | 238,8605 |
| **20 d** | 497,2312 | 632,7715 | 126,75908 | 49,16492 | 128,50136 | 120,3971 | - | 136,931 | 185,468 | 156,235 | 88,6726 | 1127,1139 | 0 | 210,6089 | 162,7925 | - | 36,80988 | 161,843 |
| **30 d** | 273,4321 | 584,4393 | 114,46801 | 27,41138 | 61,10804 | 54,70994 | - | 107,2629 | 413,4854 | 425,1786 | - | 663,6288 | 182,543 | 28,90658 | 78,31686 | 0 | 0 | 120,0335 |

|  | Minimum and maximum venom concentration (melittin and phospholipase A2) during hospitalization | |
| --- | --- | --- |
|  |  |  |
|  | Maximum venom concentration at hospital discharge | |

**(*)** Participants 301 and 303 were not evaluated.
